# Psychosocial Factors That Shape Patient and Carer Experiences of Dementia Diagnosis and Treatment: A Systematic Review of Qualitative Studies

**DOI:** 10.1371/journal.pmed.1001331

**Published:** 2012-10-30

**Authors:** Frances Bunn, Claire Goodman, Katie Sworn, Greta Rait, Carol Brayne, Louise Robinson, Elaine McNeilly, Steve Iliffe

**Affiliations:** 1 Centre for Research in Primary and Community Care, University of Hertfordshire, Hatfield, Hertfordshire, United Kingdom; 2 Social Policy Research Unit, University of York, York, United Kingdom; 3 Research Department of Primary Care and Population Health, UCL Medical School, London, United Kingdom; 4 Department of Public Health and Primary Care, University of Cambridge, Cambridge, United Kingdom; 5 Institute for Ageing and Health, Newcastle University, Newcastle upon Tyne, United Kingdom; Leiden University Medical Center, Netherlands

## Abstract

**Background:**

Early diagnosis and intervention for people with dementia is increasingly considered a priority, but practitioners are concerned with the effects of earlier diagnosis and interventions on patients and caregivers. This systematic review evaluates the qualitative evidence about how people accommodate and adapt to the diagnosis of dementia and its immediate consequences, to guide practice.

**Methods and Findings:**

We systematically reviewed qualitative studies exploring experiences of community-dwelling individuals with dementia, and their carers, around diagnosis and the transition to becoming a person with dementia. We searched PubMed, PsychINFO, Embase, CINAHL, and the British Nursing Index (all searched in May 2010 with no date restrictions; PubMed search updated in February 2012), checked reference lists, and undertook citation searches in PubMed and Google Scholar (ongoing to September 2011). We used thematic synthesis to identify key themes, commonalities, barriers to earlier diagnosis, and support identified as helpful. We identified 126 papers reporting 102 studies including a total of 3,095 participants. Three overarching themes emerged from our analysis: (1) pathways through diagnosis, including its impact on identity, roles, and relationships; (2) resolving conflicts to accommodate a diagnosis, including the acceptability of support, focusing on the present or the future, and the use or avoidance of knowledge; and (3) strategies and support to minimise the impact of dementia. Consistent barriers to diagnosis include stigma, normalisation of symptoms, and lack of knowledge. Studies report a lack of specialist support particularly post-diagnosis.

**Conclusions:**

There is an extensive body of qualitative literature on the experiences of community-dwelling individuals with dementia on receiving and adapting to a diagnosis of dementia. We present a thematic analysis that could be useful to professionals working with people with dementia. We suggest that research emphasis should shift towards the development and evaluation of interventions, particularly those providing support after diagnosis.

*Please see later in the article for the Editors' Summary.*

## Abstract 2

A systematic review of qualitative studies conducted by Frances Bunn and colleagues identifies and describes the experiences of patients and caregivers on receiving and adapting to a diagnosis of dementia.

## Editors' Summary

**Background:**

Dementia is a decline in mental ability severe enough to interfere with daily life. Alzheimer disease is the most common type of dementia. People with dementia usually have problems with two or more cognitive functions—thinking, language, memory, understanding, and judgment. Dementia is rare before the age of 65, but about a quarter of people over 85 have dementia. Because more people live longer these days, the number of patients with dementia is increasing. It is estimated that today between 40 and 50 million people live with dementia worldwide. By 2050, this number is expected to triple.

One way to study what dementia means to patients and their carers (most often spouses or other family members) is through qualitative research. Qualitative research aims to develop an in-depth understanding of individuals' experiences and behavior, as well as the reasons for their feelings and actions. In qualitative studies, researchers interview patients, their families, and doctors. When the studies are published, they usually contain direct quotations from interviews as well as summaries by the scientists who designed the interviews and analyzed the responses.

**Why Was This Study Done?:**

This study was done to better understand the experiences and attitudes of patients and their carers surrounding dementia diagnosis. It focused on patients who lived and were cared for within the community (as opposed to people living in senior care facilities or other institutions). Most cases of dementia are progressive, meaning symptoms get worse over time. Diagnosis often happens at an advanced stage of the disease, and some patients never receive a formal diagnosis. This could have many possible reasons, including unawareness or denial of symptoms by patients and people close to them. The study was also trying to understand barriers to early diagnosis and what type of support is useful for newly diagnosed patients and carers.

**What Did the Researchers Do and Find?:**

The researchers conducted a systematic search for published qualitative research studies that reported on the experience, beliefs, feelings, and attitudes surrounding dementia diagnosis. They identified and reviewed 102 such studies. Among the quotations and summaries of the individual studies, they looked for prominent and recurring themes. They also compared and contrasted the respective experiences of patients and carers.

Overall, they found that the complexity and variety of responses to a diagnosis of dementia means that making the diagnosis and conveying it to patients and carers is challenging. Negative connotations associated with dementia, inconsistent symptoms, and not knowing enough about the signs and symptoms were commonly reported barriers to early dementia diagnosis. It was often the carer who initiated the search for help from a doctor, and among patients, willingness and readiness to receive a diagnosis varied. Being told one had dementia had a big impact on a patient's identity and often caused feelings of loss, anger, fear, and frustration. Spouses had to adjust to increasingly unequal relationships and the transition to a role as carer. The strain associated with this often caused health problems in the carers as well. On the other hand, studies examining the experience of couples often reported that they found ways to continue working together as a team.

Adjusting to a dementia diagnosis is a complex process. Initially, most patients and carers experienced conflicts, for example, between autonomy and safety, between recognizing the need for help but reluctance to accept it, or between living in the present and dealing with anxiety about and preparing for the future. As these were resolved and as the disease progressed, the attitudes of patients and carers towards dementia often became more balanced and accepting. Many patients and their families adopted strategies to cope with the impact of dementia on their lives in order to manage the disease and maintain some sort of normal life. These included practical strategies involving reminders, social strategies such as relying on family support, and emotional strategies such as using humor. At some point many patients and carers reported that they were able to adopt positive mindsets and incorporate dementia in their lives.

The studies also pointed to an urgent need for support from outside the family, both right after diagnosis and subsequently. General practitioners and family physicians have important roles in helping patients and carers to get access to information, social and psychological support, and community care. The need for information was reported to be ongoing and varied, and meeting it required a variety of sources and formats. Key needs for patients and carers mentioned in the studies include information on financial aids and entitlements early on, and continued access to supportive professionals and specialists.

**What Do These Findings Mean?:**

Qualitative studies to date on how patients and carers respond to a diagnosis of dementia provide a fairly detailed picture of their experiences. The summary provided here should help professionals to understand better the challenges patients and carers face around the time of diagnosis as well as their immediate and evolving needs. The results also suggest that future research should focus on the development and evaluation of ways to meet those needs.

**Additional Information:**

Please access these websites via the online version of this summary at http://dx.doi.org/10.1371/journal.pmed.1001331.

- Wikipedia has pages on dementia and qualitative research (note that Wikipedia is a free online encyclopedia that anyone can edit)
- Alzheimer Europe, an umbrella organization of 34 Alzheimer associations from 30 countries across Europe, has a page on the different approaches to research
- The UK Department of Health has pages on dementia, including guidelines for carers of people with dementia
- MedlinePlus also has information about dementia

## Introduction

Dementia affects one in 20 people over the age of 65 and one in five over the age of 80. World-wide there are an estimated 35.6 million people with dementia. By 2050 this number will rise to over 115 million [1]. In 2010 the total estimated worldwide costs of dementia were US$604 billion, with 70% of the costs occurring in western Europe and North America [2]. There is evidence that many patients who meet the criteria for dementia never receive a formal diagnosis [3]–[6] or receive a diagnosis only late in the disease trajectory [7]. There remains wide variability in current practice and attitudes to diagnostic disclosure [8], with some professionals worried about the possible harm of early diagnosis of a condition widely seen as untreatable and life-changing [9]. There is, however, growing support for early diagnosis [10]–[12], as it may improve quality of life for patients and carers, delay or prevent care home admissions [13],[14], and facilitate referral to specialist services and treatment [11],[15].

There is increasing recognition of the importance of systematically reviewing qualitative research [16], as it allows the development of in-depth understanding of persistent themes, explores transferability, and prevents unnecessary duplication of research. Previous reviews have looked at patient and carer experiences of dementia [17],[18] and disclosure of diagnosis [8],[19],[20], but none, to our knowledge, has performed a comprehensive thematic synthesis of qualitative studies exploring the experiences of people with dementia and their family members of receiving and adapting to a diagnosis, and of service delivery. Our aim was to inform the debate about early diagnosis and service provision by systematically reviewing qualitative literature on the psychosocial factors that shape patient and carer experiences of diagnosis and early treatment. We aimed to identify the following: key themes, commonalities, and differences across groups; barriers to early diagnosis; and which support services individuals newly diagnosed with dementia and their carers perceive as helpful.

## Methods

### Selection Criteria

We included qualitative studies that explored patient and carer experiences around diagnosis and treatment of dementia or mild cognitive impairment (MCI). This included studies from any established qualitative tradition and using any recognised qualitative methodology. Mixed method studies were included if they had a qualitative element, but only the qualitative data were used in the analysis. The main outcomes of interest were patient and carer attitudes, beliefs, and feelings around becoming or caring for an individual with dementia. In particular we searched for data on responses to early signs of dementia, receiving and adapting to a diagnosis, and experiences of post-diagnosis support. We focused on community-dwelling participants and excluded studies in long-term care settings or published in languages other than English. The reporting of the review follows PRISMA guidance (Text S1), and the methods for the review were pre-specified in a protocol (Text S2).

### Search Strategy

We searched for all potentially relevant published and unpublished literature, with no date restrictions, and regardless of country of origin. Studies were identified by computerised searches of PubMed (1950–2012), PsychINFO (Ovid) (1806–2010), Embase (Ovid) (1980–2010), CINAHL (EBSCO Publishing) (1980–2010), and the British Nursing Index (NHS Evidence) (1985–2010). An example search query is given in Box 1. In addition, we employed extensive lateral search techniques (ongoing March 2010–September 2011), such as checking reference lists, performing key word searches in Google Scholar, contacting experts, and using the “cited by” option in Google Scholar and the “related articles” option in PubMed. Such lateral search strategies have been shown to be particularly important for identifying non-randomised studies [21]. The original electronic database searches were conducted between March and May 2010, with the PubMed search updated in February 2012.

**Box 1. Example Search Query**

The following search query was used for the PubMed searches* (May 2010, updated February 2012):

(disability OR disablement OR aware OR awareness OR self OR fear OR emotions OR “self concept” OR self assessment OR “self care” OR adaptation OR stress OR autonomy OR denial OR sick role OR coping OR cope OR patient participation OR self disclosure OR life [ti] OR live [ti] or living [ti]) AND (confusion OR memory loss OR “early dementia” OR “mild dementia” OR “moderate dementia” OR alzheimers OR mild cognitive disorder OR (MCI (cognition disorder AND (mild OR moderate OR early)))) AND (caregivers OR social support OR self-help groups OR relatives OR carers OR health care staff OR spouses OR dyadic OR partner OR communication OR nursing)

*Search terms were adapted for other databases.

### Data Extraction and Critical Appraisal

Two reviewers independently screened titles and abstracts identified by the electronic search, applied the selection criteria to potentially relevant papers, and extracted data using a standardised checklist. Where results of a study were reported in more than one publication, we grouped reports together and marked the publication with the most complete data as the primary reference; the other papers describing the same study were classified as associated papers. We collected data on study design (including theoretical framework), aims, methods, participant characteristics, areas covered (e.g., symptom recognition, receiving a diagnosis, adjusting to a diagnosis, and issues relating to service delivery), and common themes.

Two reviewers independently assessed study quality using a checklist based on Spencer et al.'s framework for assessing quality in qualitative research [22]. This framework has been adapted by one of the authors and used in previous work [23],[24]. In addition, the overall reliability and usefulness of the study to the research questions was graded as low, medium, or high. Reliability related to the quality of the study, and usefulness to the relevancy of a paper in the context of our review. The core quality assessment principles are summarised in Table 1. Data compiled by the two reviewers were compared for agreement, and any discrepancies were resolved by discussion or by consultation with a third researcher. As there is no consensus or empirically tested method for excluding qualitative studies from reviews on the basis of quality, we included all studies regardless of their quality.

**Table 1:** Core principles of study quality assessment.

Quality Criterion	Further Details
Scope and purpose	E.g., clearly stated question, clear outline of theoretical framework
Design	E.g., discussion of why particular approach/methods chosen
Sample	E.g., adequate description of sample used and how sample was identified and recruited
Data collection	E.g., systematic documentation of tools/guides/researcher role, recording methods explicit
Analysis	E.g., documentation of analytic tools/methods used, evidence of rigorous/systematic analysis
Reliability and validity	E.g., presentation of original data, how categories/concepts/themes were developed and were they checked by more than one author, interpretation, how theories developed, triangulation with other sources
Generalisability	E.g., sufficient evidence for generalisability or limits made clear by author
Credibility/integrity/plausibility	E.g., provides evidence that resonates with other knowledge, results/conclusions supported by evidence
Overall weight for reliability/trustworthiness	Low = one or more “not at all” values for the first five criteria above, medium = at least 4/5 of the first five criteria above marked as “fully or mostly”, high = all of the first five criteria above marked “fully or mostly” and none marked “not at all”
Overall weight for usefulness of findings for review	Considers the following: (i) to what extent does the study help us to understand one or more of the topics covered in the review? (ii) how rich are the findings? (iii) has the study successfully enhanced our understanding of a new area/sample or enriched an old one?

### Analysis

We synthesised our study findings using thematic analysis. This process, which involves identifying prominent or recurring themes, has previously been used to successfully synthesise a large number of studies [25], and draws on existing literature around the synthesis of qualitative research [26]–[29]. Defining what counts as “data” in qualitative research is not straightforward [29]. We took the approach suggested by Thomas and Harden [29] and took “data” to be not just that in the form of quotations but all of the text labelled as “results” or “findings” in study reports.

Studies judged to have met the inclusion criteria were independently reviewed and coded by two reviewers by hand. They applied open codes to text, identified themes, and documented supporting evidence in the form of quotes. From this, a list of initial codes and themes was created. These codes were then inserted into qualitative analysis software (NVivo), and PDFs of all included studies were imported for further analysis. The use of such software can facilitate the development of themes and allow reviewers to examine the contribution made to their findings by individual studies or groups of studies [29]. Analysis in NVivo involved examination of concepts for similarities and differences, refinement of descriptive themes, verification that data was a “good fit” for the themes, and development of analytic themes. We then undertook a final process of verification using the 28 studies that had scored high for both reliability and usefulness.

### Ethical Approval

Ethical approval was not required for this work.

## Results

### Description of Studies

**Figure 1:**
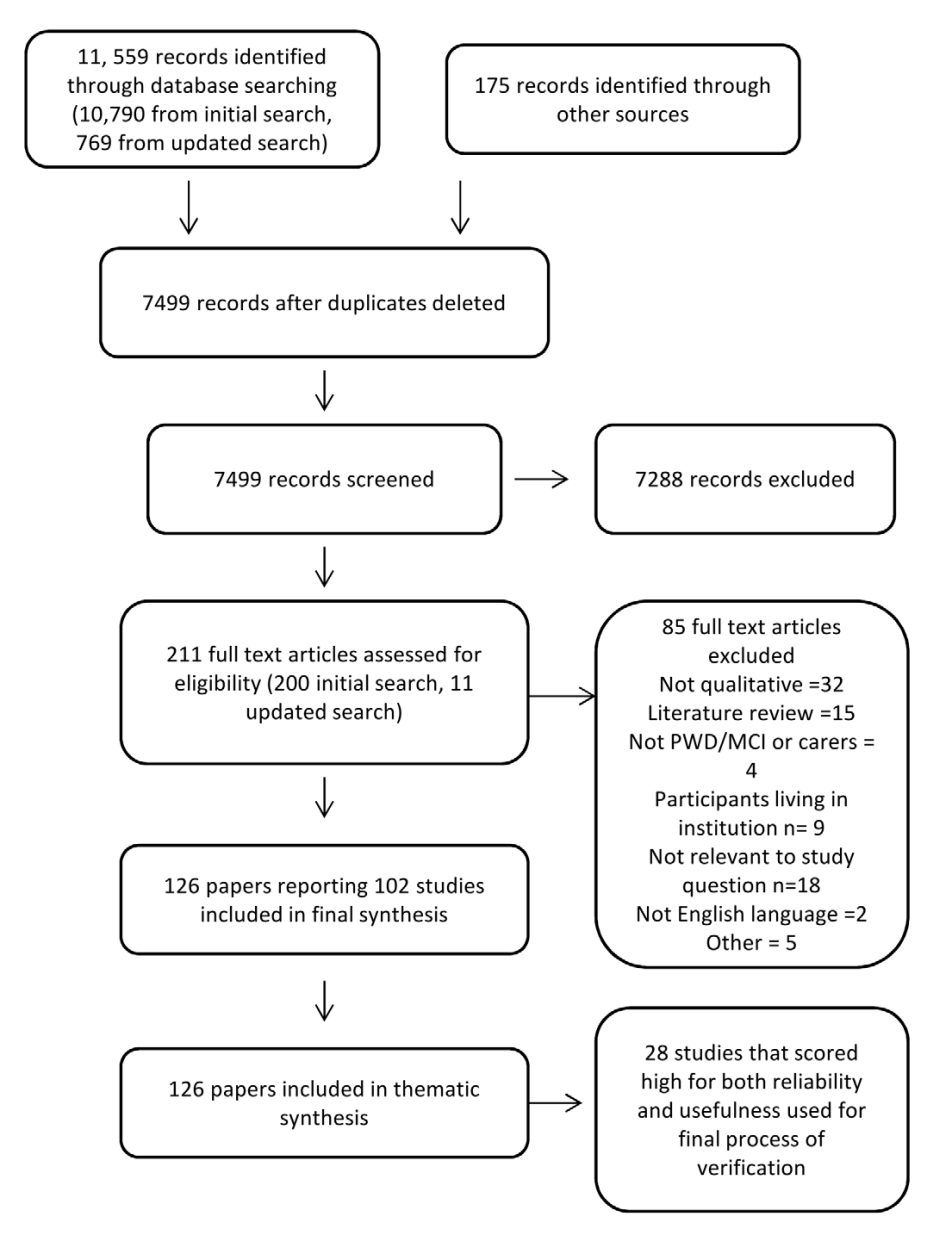
Flow chart of study selection process. PWD, person with dementia.

In all, 126 papers met our inclusion criteria. Of those, 102 were classified as primary studies [30]–[131], and a further 24 as associated papers [132]–[155]. The links between primary and associated papers can be seen in Table S1. An overview of the selection process can be seen in Figure 1.

Studies included a total of 3,095 participants: 1,145 individuals with dementia or MCI and 1,950 informal carers. We found studies conducted in 14 different countries, although the majority (76%) were from the UK or North America. Study characteristics are summarised in Table 2, with details of individual studies provided in Table S1. Participants were community-dwelling, the majority lived with a carer, and they were predominantly white. However, 16 studies [32],[42],[66],[67],[70],[72],[74],[85],[86],[88],[92],[98]–[100],[118],[122] either focused on the views and experiences of black and minority ethnic groups in the UK and North America or compared the views of different ethnic groups.

**Table 2:** Overview of study characteristics.

Study Information	Study Methods	Type of Participants
**Year of publication**	**Data collection methods**	**Participants**
Range 1989–2011, half published from 2005 onwards	Some used more than one approach	PWD *n* = 61
**Country**	Most common methods: interviews *n* = 93, focus groups *n* = 18	Person with MCI *n* = 13
UK *n* = 41	**Methodological approach**	Informal carers of PWD/person with MCI *n* = 72
US *n* = 27	Phenomenological *n* = 29	**Age**
US and UK *n* = 2	Ethnographic *n* = 5	Range 40–97 y, but majority over 70 y
Europe (excluding UK) *n* = 16	Grounded theory *n* = 27	Of 35 studies that gave mean/median age, majority had mean age in the 70 s
Canada *n* = 11	Other *n* = 8 (e.g., biographical approach, case study)	**Ethnicity**
Rest: Australia (*n* = 1), New Zealand (*n* = 1), and Asia (*n* = 3)	Not specified *n* = 33	Not specified *n* = 41
**Subject areas covered in study** a	**Recruitment**	White participants only *n* = 27
Symptom recognition *n* = 32	Most commonly recruited from: memory clinics *n* = 38, voluntary organisations *n* = 23	Asian participants only *n* = 7
Receiving diagnosis *n* = 37	**Sample**	Black participants only *n* = 1
Adjusting to diagnosis/condition *n* = 78 (includes PWD and carer perspective)	Convenience sample *n* = 35	Mixture of ethnic backgrounds *n* = 26
Service delivery *n* = 25	Purposive or theoretical sample *n* = 63	**Received a diagnosis**
**Setting**	Not clear *n* = 4	Yes *n* = 74
Community-dwelling	**Number of data collection points**	No *n* = 4
Living with family member *n* = 46	One *n* = 73	Rest mixture or not clear
In other studies, the majority lived with family member (most commonly a spouse)	Two *n* = 10	**Stage of dementia**
	More than two *n* = 13	26 studies reported MMSE or similar, all but two mild/moderate
	Different for different participants *n* = 6	**Type of dementia**
		Not specified *n* = 42
		Where it was reported most common type was Alzheimer disease *n* = 53, early onset *n* = 3

Sample sizes refer to the numbers of studies, not the number of individual participants.

a Studies sometimes classified as more than one category.

MMSE, Mini-Mental State Examination; PWD, person with dementia.

### Study Quality

**Figure 2:**
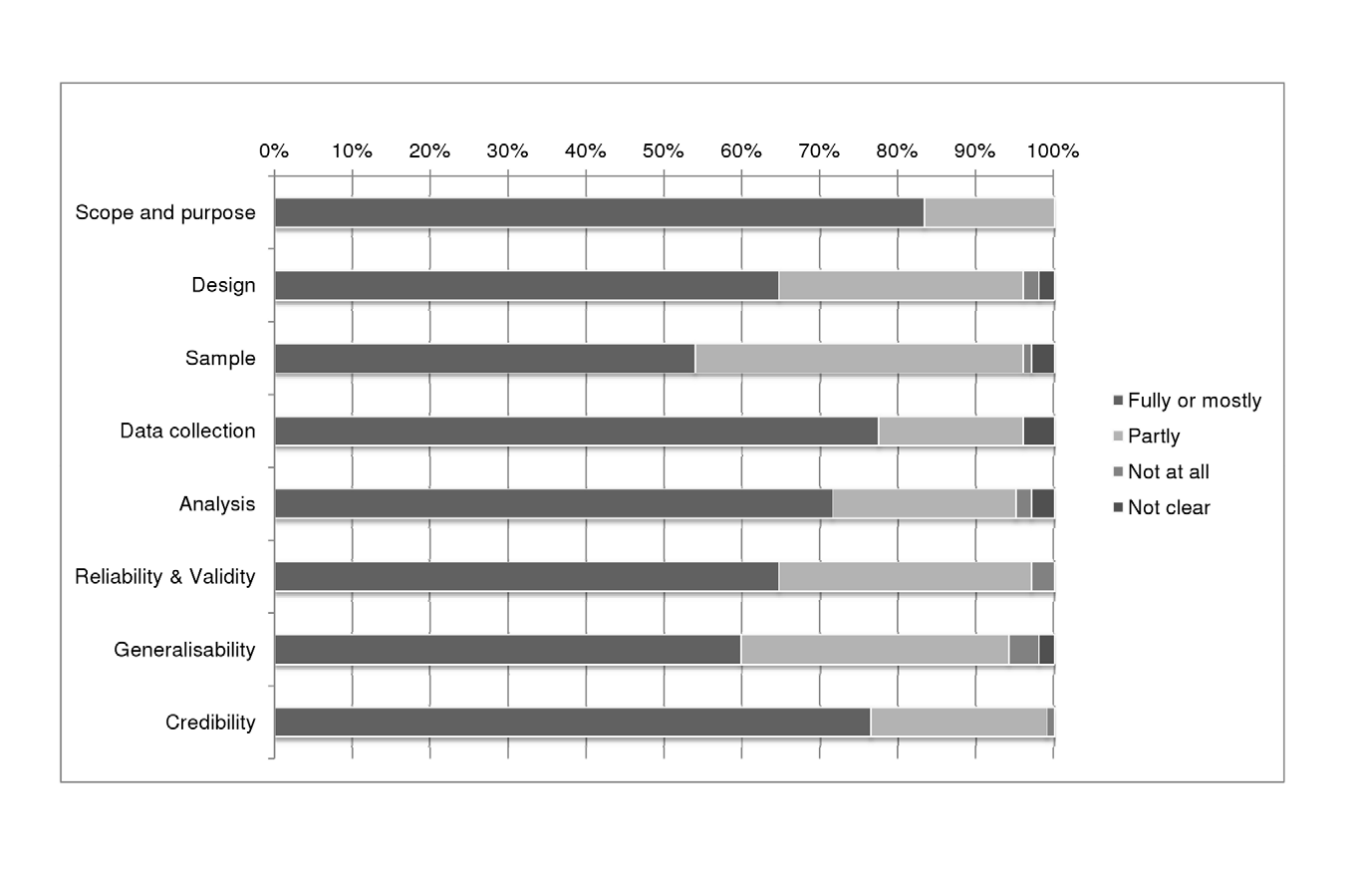
Summary of quality assessment domains. This figure shows review authors' judgements about each quality domain presented as percentages across all included studies.

Overall, 32% of the studies scored high for reliability, 32% medium, and 35% low, and 57% scored high, 30% medium, and 13% low for usefulness; 27% scored high for both reliability and usefulness. A summary of individual quality assessment scores can be found in Table S1 and of overall quality assessment domains in Figure 2.

### Findings from the Thematic Analysis

**Figure 3:**
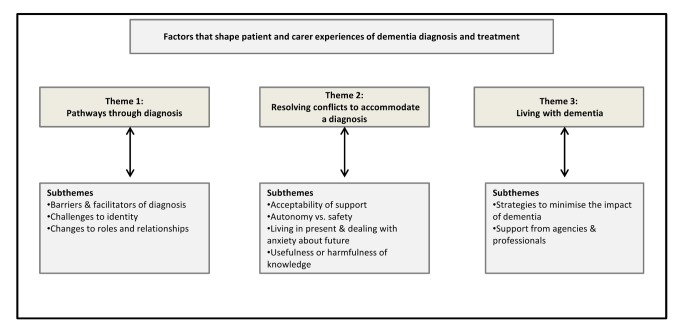
Themes and subthemes. This figure shows the three overarching themes and the related subthemes that emerged from our analysis.

We identified three overarching thematic categories as being central to the process of receiving and adapting to a diagnosis of dementia: (1) pathways through diagnosis (including barriers and facilitators of earlier diagnosis, and the challenge of the diagnosis to identity, roles, and relationships); (2) conflicts that need to be resolved to accommodate the diagnosis (including the acceptability or otherwise of support, autonomy versus safety, the need to focus on today or tomorrow, and the usefulness or harmfulness of knowledge); and (3) living with dementia (including practical strategies to minimise the impact of dementia, and the support that professionals and agencies can give) (see Figure 3). The evidence to support these themes can be seen in Table S2.

The themes apply both to the individuals with dementia and to their family carer/s, and are not necessarily sequential but are interlinked and reflect ongoing processes of adjustment that can occur from the moment symptoms of dementia appear. Quotes supporting each theme are presented in Table S3. Each thematic category is discussed in more detail below. Supporting citations in the text are representative rather than comprehensive.

### Theme 1: Pathways through Diagnosis

#### Barriers to early diagnosis

Among our sample, persistent barriers to early diagnosis were stigma, the normalisation of symptoms, and a lack of awareness about the signs and symptoms of dementia. It often took a trigger event or tipping point such as a hospitalisation or bereavement [34],[37],[40],[42],[51],[53],[92],[114],[118],[138] before people sought help. Family members often recognised something was wrong before the person with dementia did, and were frequently instrumental in obtaining a diagnosis [32],[40],[41],[45],[54],[72],[88],[115],[129]. There was evidence of greater stigma among minority ethnic populations and evidence that they were less likely to recognise symptoms of dementia as an illness than white individuals, and more likely to ascribe these symptoms to the ageing process [32],[86],[118],[119]. In addition, symptoms of dementia were sometimes given cultural or religious explanations [118]. As many studies did not provide detailed demographic information, it was unclear whether level of education or socioeconomic status impacted on awareness of dementia and help-seeking behaviour. Studies suggest that in some cases doctors are slow to recognise symptoms or reluctant to give a diagnosis [38],[39],[68],[74],[88],[92],[124], and that even when people have been referred to memory services the process may be slow, with long periods of waiting [93],[152].

#### Impact of diagnosis

Regardless of culture and context, we found many similarities in individual's experiences of becoming a person with dementia. Dementia had an enormous impact on identity [72],[91],[121], leading to feelings of loss, anger, uncertainty, and frustration. [33],[37],[50],[53],[58]–[61],[77],[79],[109]. People with dementia struggled to preserve aspects of their former self and were often supported in this by family carers, who focused on remaining abilities rather than drawing attention to mistakes [109],[144]. Dementia also had a significant impact on roles and relationships, both within the family and in wider social networks. A desire to preserve a pre-dementia identity sometimes led to people being reluctant to disclose their diagnosis to family or wider social networks [52],[53],[109], which could lead to social isolation [53],[62],[105]. Despite this, some studies suggested that eventually both the individuals with dementia and their carers reached a state of acceptance [36],[45],[88],[91].

Unsurprisingly, our analysis revealed that dementia had a significant impact on both the individuals with dementia and their families. Spouses had to adjust to increasingly unequal relationships [30],[54],[63],[64],[97],[104],[114],[123],[125], and communication between the couple was often affected. However, studies looking at the experiences of couples often found an emphasis on working together as a team, with a high degree of mutuality [39],[50],[88],[91],[110],[134]. It was clear that there was often significant strain on carers [39],[42],[44],[56],[109],[119],[123],[127], which often impacted adversely on their own health [43],[44],[61],[78],[117].

For some individuals, receiving a diagnosis was the beginning of the adjustment process, but for others, who had been experiencing symptoms for some time, considerable adjustment had already preceded the diagnosis. Although diagnosis was often traumatic [38],[50],[51],[62],[152], the validation of suspicions could come as a relief [45],[93],[129]. There was also a proportion of individuals with dementia and carers who continued to consider memory loss insignificant even after diagnosis [51],[93],[119].

### Theme 2: Resolving Conflicts to Accommodate a Diagnosis

Among our sample, beliefs about, and perceptions of, dementia varied considerably, with meanings attached to a diagnosis being shaped by the individual's current situation, by past experiences, and by exposure to others with dementia. For example, families that included a member with professional experience of dementia or who had a relative with dementia were more likely to completely acknowledge a diagnosis than those who had had no previous exposure to dementia [84]. Adjusting to a diagnosis is a complex process, and a number of ambiguities and polarised findings emerged from our analysis (see Box 2). These ambiguities represent conflicts that may need to be resolved in order to accommodate a diagnosis.

**Box 2. Polarised Findings**

- Reactions to, and readiness for, a diagnosis may vary between individuals and between the individuals with dementia and their family/carers.
- Individuals with dementia and their families/carers may struggle to preserve a pre-dementia identity whilst also adapting to a diagnosis and assimilating the disease into a new identity.
- Carers may be torn between protecting the person with dementia and promoting their independence.
- Individuals with dementia and their carers may focus on the present whilst also experiencing anxiety about the future.
- There can be a tension between a desire to maintain social contacts and strategies to minimise or normalise the impact of dementia.
- Information may be empowering for some people, but others may reject new knowledge and resist a diagnosis.
- Peer support is often beneficial but can have a negative impact by showing what the future may hold.

Studies identified the potential conflict that could arise as people strove to preserve identity and autonomy in the face of increasing symptoms. This sometimes led to an apparent unawareness of or a resistance to acknowledge a diagnosis [37]. Study authors did not, however, interpret this simply as denial but rather as a self-maintaining strategy [136] or a deliberate choice to be seen as an agent rather than an object [90]. This was reflected in individual's attitudes towards information, and there was evidence that some people with dementia and their carers actively sought information [89],[90], whereas others rejected new knowledge. However, understanding of, and attitudes towards, dementia were not fixed, and evolved throughout the disease trajectory.

### Theme 3: Living with Dementia

This theme relates to the strategies that individuals with dementia and their families adopted to deal with the impact of dementia on their lives, and also to the support they required from professionals and agencies. The adoption of strategies to manage the disease, minimise losses, reduce social isolation, and maintain normalcy was common [50],[58],[69],[90],[134]. This included practical strategies such as using reminders or prompts, social strategies such as relying on family support, and emotional strategies such as using humour or finding meaningful activity.

### Supporting People with Dementia and Their Carers

The general practitioner or family physician was generally the first point of contact for people with dementia and their carers [43],[93] and had an important role to play in facilitating service access [43],[74]. However, in our sample, experiences were mixed, with some participants reporting a delay in referral to memory services and others reporting confidentiality obstacles, with doctors reluctant to talk to carers about their family member with dementia [74],[88],[106],[124]. Attending memory clinics could be shocking or frightening [51],[75],[94], and receiving a diagnosis could lead to increased tension as someone negotiated a new identity as a person with dementia [37].

There was a clear need for greater support after diagnosis [78], including advice [61],[129], social and psychological support [61],[125], access to community care [43],[93], and respite [127]. There was evidence that valuable support was provided by voluntary organisations such as the Alzheimer's Society [93],[106],[114], although signposting to these needed to be improved [61]. Information provision was seen as key in many studies, but it was clear that better knowledge sharing at point of diagnosis was not always the solution [152]. The information needs of patients varied over time, and information provision needed to be ongoing, with flexibility in timing and format [93],[106].

Amongst people with Alzheimer disease and their family carers, there was variation in perceptions of the benefits of acetylcholinesterase inhibitors, with some studies reporting that medication gave people hope [48],[71],[97],[111],[121],[152], one study reporting that patients and their carers felt that the benefits were not clear but they were “worth a try” [71], and two studies reporting that patients felt medication had little to offer them [62],[106]. Studies suggested that there was now an increasing expectation that medication would be available [93], that attitudes towards medication got less positive over time [49], and that drug treatment was often initiated by the carer [71].

Amongst our sample it was clear that many people found peer support valuable [37],[38],[72],[93],[129],[131]. However, for others there could be negative consequences, as the inclusion of people at different stages in the dementia trajectory could make people aware of what the future held for them [37]. The timing of referral to community-based support groups may be key [43],[78],[106], and such decisions are likely to be facilitated by continuous therapeutic relationships between individuals with dementia and the practitioners involved in their care.

## Discussion

We found 102 studies exploring the experiences of community-dwelling individuals with dementia or MCI, and their family carers, of diagnosis, treatment, and the transition to becoming a person with dementia. What emerged from our analysis was the complexity and variety of responses to becoming a person with dementia, and how this makes diagnosing and supporting this group particularly challenging. There were many commonalities, but beliefs and experiences were context-specific and could be polarised. For example, willingness or readiness to receive a diagnosis varied, and there was evidence that it was often a carer rather than the person with memory problems who initiated a diagnosis. Carers were also often torn between protecting the person with dementia and promoting his or her independence. Moreover, it was clear that individuals with dementia and their family and friends were simultaneously struggling to preserve a pre-dementia self whilst at the same time accommodating the diagnosis and assimilating the disease into a new identity.

We used systematic and rigorous methods for reviewing qualitative literature. However, there are a number of methodological issues that could affect the validity of our findings. Qualitative studies are challenging to identify using standard search techniques [156],[157], and despite our efforts to identify all available studies, we cannot exclude the possibility that some were missed. Moreover, excluding studies reported in languages other than English may have introduced bias. However, we used a comprehensive search strategy, including extensive lateral searching to minimise missing studies, we included studies from 14 different countries, and we are confident that we have reached thematic saturation.

We did not exclude studies from our review on the basis of quality, but we did attempt to “weight” studies by using only those that scored high for both reliability and usefulness in a final process of verification of our themes. However, this approach may be contentious, as there is no consensus on what constitutes a “good” quality qualitative study, nor well-established methods for weighting qualitative studies [22],[158]. That said, our quality assessment procedures were thorough, and the process of reaching inter-reviewer agreement maximised robustness.

Although not all studies provided demographic information, analysis of the characteristics of participants in the studies included in the systematic review suggested that there was a skew towards more affluent, educated participants, most of whom were white. Whilst qualitative research does not generally set out to be representative, it is appropriate to consider the transferability of findings. It is, therefore, a concern that much of the research in this area has been carried out on those populations more easily accessible to researchers. Such populations may have different attitudes to information provision and be more accustomed to self-advocacy [37]. Moreover, although more than 40% of studies did not specify what type of dementia participants had, where this information was given, it was clear that the majority had Alzheimer disease. The experiences of individuals with Alzheimer disease and their carers may not be directly transferable to people with other types of dementia.

The themes in the review relating to coping strategies, the impact of dementia on quality of life and relationships, and experiences of care were similar to those from other reviews [17],[18]. It has been suggested that dementia is not necessarily a source of dreadful suffering [18],[19], and, indeed, among our sample we found evidence that many people adopted positive mindsets and appeared to successfully incorporate dementia into their lives. Nevertheless, it was clear that dementia has a significant impact on people's lives and relationships and is a major threat to identity. Moreover, the progressive nature of the disease means that the process is cyclical and requires constant adjustment. This review highlights how priorities and views change over time, and the need for services to be organised to address that process. Previous reviews [8],[19] have found that people with dementia and their carers are generally in favour of disclosure, and this is supported by this review. However, it is clear that a tension exists between the self-maintaining strategies people employ to minimise the impact of dementia on their lives and sense of self, and the acceptance of a diagnosis and all its implications [136].

### Implications for Practice

Our review suggests that key needs for people with dementia and their carers include the early provision of information about financial aids and entitlements, the opportunity to talk to supportive professionals, signposting to appropriate statutory and voluntary services, and specialist support. Support needs to be ongoing, flexible, and sensitive to the needs of different groups, such as those with early onset dementia [38] or minority ethnic groups [118]; it needs to take into account the needs for continuity of care [33]; and it needs to manage care needs and safety whilst being aware of the person's sense of identity and dignity [125]. In the UK it has been suggested that specialist dementia advisors might provide such support [159]. Indeed, it is clear from the literature that the needs of people with dementia and their carers are complex and varied, and those making decisions about the timing and delivery of services need appropriate expertise and training. A further consideration relates to the availability of appropriate resources. It is possible that publicity around early diagnosis, such as the UK government campaign to raise public awareness of the early signs of dementia, may be raising expectations of services in the earlier stages of the illness [93]. This has implications for service delivery, as it may lead to the diversion of resources away from those with more advanced dementia.

### Implications for Research

This review provides a comprehensive account of studies reporting the experiences of community-dwelling individuals with dementia and their carers on receiving a diagnosis and becoming a person with dementia. Indeed, there is now a substantial body of qualitative research on the transition to becoming a person with dementia. However, such research has largely been carried out in community-based populations that are easily accessible to researchers. Less is known about the oldest old, those who do not access services, or those have comorbid health conditions. Our review excluded individuals living in long-term care, and further research may be needed to explore issues around diagnosis for people living in residential homes. Although we included experiences of post-diagnosis support and treatment, the focus of our review was on the earlier stages of dementia, and we did not address issues such as behavioural problems or the transition to long-term care. Furthermore, many studies provided little or no demographic data, and it was difficult to assess the impact of factors such as type of dementia, level of education, or other socioeconomic factors on patient and carer experiences. Future qualitative studies should consider including greater detail about the characteristics of participants so that transferability can be better assessed.

### Conclusions

It is often suggested that the voice of the person with dementia is not present in research. We found this not to be the case in qualitative research studies. There is now a substantial body of qualitative evidence relating to the experiences of community-dwelling individuals with cognitive impairment and their family carers, particularly in relation to the transition to becoming a person with dementia. This review provides a comprehensive account of how people accommodate and adapt to a diagnosis of dementia that could be useful to professionals working with individuals with dementia. The synthesis focuses attention on three aspects of the diagnostic transition: the challenge the diagnosis poses to identity and role, the conflicts that may need to be resolved to accommodate the diagnosis, and the practical management strategies that can assist individuals with dementia and their families. The next steps to ensure patient benefit should involve the development and evaluation of interventions, particularly those relating to post-diagnosis support.

## Supporting Information

Text S1 **PRISMA checklist.**
(DOC)

Text S2 **Protocol for the review.**
(DOC)

Table S1 **Characteristics of included studies.**
(DOCX)

Table S2 **Themes and supporting evidence.** This table shows the themes that arose from our analysis and the evidence to support them.
(DOCX)

Table S3 **Examples of quotations illustrating themes and author interpretations of findings.** This table provides examples of quotes supporting the themes from our analysis and gives examples of the ways authors of our included studies interpreted their findings.
(DOCX)

## Abbreviations

MCI: mild cognitive impairment
