# Supplementary material for: Psychosocial Factors That Shape Patient and Carer Experiences of Dementia Diagnosis and Treatment: A Systematic Review of Qualitative Studies
Source: PLoS Med. 2012 Oct 30;9(10):e1001331. doi: 10.1371/journal.pmed.1001331 (PMC3484131; doi:10.1371/journal.pmed.1001331)
Supplement: Text S2 — Protocol for the review. (DOC) [file pmed.1001331.s002.doc]

EXPLORE DD – SYSTEMATIC REVIEW PROTOCOL

An exploration of patient experiences around diagnosis and treatment of dementia: implications for service development:

1. BACKGROUND

It has been estimated that over 700,000 people in the UK have dementia, the most common form being Alzheimer’s disease (Alzheimer's Society, 2007). Although there are significant differences in the physical and cognitive effects of the different types of dementias all are progressive, involve increasing physical and mental deterioration, and lead to a sufferer becoming increasingly dependent. It is estimated that the number of people with dementia in the UK will double to 1.4 million in the next 30 years (Alzheimer's Society, 2007) with an estimated cost of £40 billion (Kings Fund, 2008).

Despite the prevalence of dementia there is evidence that many patients who meet the criteria for dementia never receive a formal diagnosis (Iliffe et al., 1990, Boustani et al., 2003, National Audit Office, 2007, Banerjee and Chan, 2008) or that a diagnosis is only received late in the trajectory of the illness when opportunities for intervention are more limited (Dening and Milne, 2008). There remains wide variability in current practice and attitudes to diagnostic disclosure (Bamford et al., 2004) with some professionals worried about the possible harm of early diagnosis of a condition widely seen as negative and life-changing (Iliffe et al., 2003). There is, however, a growing consensus that a diagnosis of dementia should be made as early as possible (NICE, 2007, National Audit Office, 2007) as it may improve quality of life for patients and carers, delay or prevent care home admissions (Banerjee et al., 2007, Mittelman et al., 2007) and facilitate referral to appropriate specialist services (Lingard and Milne, 2004, National Audit Office, 2007). Early diagnosis and intervention is a key theme of the forthcoming Dementia Strategy.

Qualitative research allows us to describe and explain behaviour in its context and there is a growing recognition of the importance of systematically reviewing qualitative research (Dixon-Woods and Fitzpatrick, 2001). In a recent systematic review in the Lancet researchers reviewed over 200 studies of young people’s sexual behaviour (Marston and King, 2006). This review, and others like it, have demonstrated that synthesising multiple small qualitative studies allows the development of in-depth understanding of persistent themes, explores transferability and prevents unnecessary duplication of research. This project will apply methods for the synthesis of qualitative research to establish an evidence base for medical and nursing practice when identifying and supporting people newly diagnosed with dementia.

Research exploring perspectives of becoming a patient with dementia are done in a wide range of settings. It is likely there are elements and themes that transcend culture and context and that can inform how clinicians and services respond to patients at this key time of transition. Other literature reviews have looked at the patient experience of dementia (Steeman et al., 2006, Lecouturier et al., 2008, Milne and Peet, 2008) and diagnosis (Bamford et al., 2004). However, gaps in the evidence base remain and there is a need for more information on views of service barriers and facilitators and the needs of different cultural groups. This systematic review, with additional stakeholder involvement, including representatives from BME groups, will examine these issues and include an in-depth exploration of how the findings can directly inform the debate about early diagnosis and appropriate service provision. It will contribute to the development of guidance for health care professionals.

2. AIMS OF THE PROJECT

This study aims to systematically review the international qualitative literature on the way people, and their carer’s, experience the process of transition to becoming a person with dementia. This will include how they became aware of early symptoms of dementia and will focus on their experiences of receiving and adapting to a diagnosis of dementia. In particular we are interested in the barriers and facilitators to early diagnosis and treatment. Including the international literature will enable us to examine how issues such as culture and ethnicity affect this process.

The overarching research questions are:

- What are the key themes emerging from the literature and what commonalities and differences exist across different groups and cultures?
- What are the barriers/facilitators to early diagnosis and service provision?
- What kind of support and interventions from primary and hospital care services do people newly diagnosed with dementia and their carers perceive as helpful and unhelpful?
- Are there common experiences and responses to early signs of dementia and can these be used to inform the development of services for people newly diagnosed with dementia?

3. RESEARCH PLAN AND METHODOLOGY

Previous reviews have demonstrated that synthesising qualitative research allows greater understanding and the attainment of conceptual and theoretical development beyond that achieved in a single study (Campbell et al., 2003, Marston and King, 2006, Thomas and Harden, 2008).

3.1 Systematic Review Methods

3.1.1 Inclusion criteria

The review will include studies that explore issues around becoming a person with dementia. For example, at what stage and how did they come into contact with health care services, what factors influenced how the diagnosis was communicated, how did receiving a diagnosis affect the way in which they responded to, and coped with, the condition, what were the aspects of care and service provision that they found most helpful and unhelpful.

Types of studies

The aim of the review is to provide an in-depth exploration of the personal beliefs and experiences of individuals as they undergo the transition to becoming a person with dementia. We will include all relevant qualitative studies that use recognised methods for qualitative research.

Types of participants

Although the majority of studies are likely to involve older people we will include studies that involve people of any age and with all types of dementia, apart from CJD and HIV related dementia (they are excluded because the type and course of the illness is different from other types of dementia). The focus will be particularly on early-stage dementia where people start to become aware of symptoms and may present to health care services. We will also include relevant studies looking at the experiences of the informal carers of people with dementia.

Settings

We will include studies involving community dwelling participants. This will include people in sheltered housing and assisted living who rely on primary care services but will exclude those in long-term care.

Outcomes

The main outcomes will be patient and carers attitudes, beliefs and feelings around becoming a person with dementia. In particular we will look at: responses to early signs of dementia, receiving a diagnosis and experiences of service provision. This will include data on barriers and facilitators to diagnosis and service provision and perceptions of what is helpful and unhelpful.

3.1.2 Identification of studies

Qualitative studies can be particularly challenging to identify using standard search techniques (Wong et al., 2004, Barroso et al., 2003). This may be because there are no well-established methodological filters and MeSH headings and index terms have not been applied as consistently and appropriately as for other types of research. In addition, many of the issues we are interested in examining may be embedded in papers exploring experiences associated with dementia more generally. Our search strategy will, therefore, be designed to be broad and sensitive enough to ensure that we capture all potentially relevant studies. This will include a highly sensitive electronic search strategy and the employment of other lateral searching techniques which have been shown to be important for identifying non randomised studies (Greenhalgh and Peacock, 2005). The electronic search strategy will be developed by an experienced Information Scientist (Reinhard Wentz) with input from the rest of the project team. We will search for English language published and unpublished literature. There will be no date restrictions on the searches. Searches will include:

- Electronic databases including: Medline (PubMed), CINAHL, BNI, EMBASE, PsycInfo, DH Data, Kings Fund, Web of Science (WoS incl. SCI, SSCI, HCI), TRIP, Cochrane Library ( incl. CENTRAL, CDSR, DARE, HTA), AgeInfo (Centre for policy on Ageing – UK), NTIS, SIGLE.
- Checking of reference lists from primary studies and systematic reviews (snowballing) (Dixon-Woods, 2006)
- Citation searches using the ‘Cited by’ option on WoS, Google Scholar and Scopus, and the ‘Related articles’ option on PubMed and WoS (‘Lateral Searching’) (Greenhalgh and Peacock, 2005)
- Contact with experts and those with an interest in this field to uncover grey literature (e.g. DeNDRoN, National Library for Health Later Life Specialist Library, Alzheimer’s society and For Dementia)

On PubMed relevant MeSH terms will be automatically identified and used for the search. Where possible, cross-database *test* searches will be run on services which cover a range of databases (such as OVID range of databases or The National Library of Health) to identify resources which are likely to yield substantial numbers of studies relevant to this review.

3.1.3 Study Screening

Electronic search results will be downloaded into EndNote bibliographic software and, where possible, duplicates deleted. As there is evidence that two reviewers should screen records to maximize ascertainment of relevant studies (Edwards et al., 2002) two reviewers (FBunn and the RA) will independently screen titles and abstracts against the predefined inclusion criteria. Full manuscripts of all potentially relevant citations will be obtained. Hard copies will then be screened independently by two reviewers using a screening form with clearly defined criteria. Any disagreements will be resolved by consensus or by discussion with a third author.

3.1.4 Data Extraction and Critical Appraisal

For studies that meet the inclusion criteria data will be extracted onto a pre-designed, and piloted, form. As independent double data extraction is considered to reduce the risk of error (Higgins et al 2008) data will be extracted independently by two reviewers. Data will be entered into an Access database. Data extracted will include:

- Type of study design (e.g. theoretical framework)
- Study aims/research question
- Methods used (e.g. questionnaires, semi-structured interviews, in-depth interviews, focus groups)
- Type of participants (including age, type of dementia, sex, ethnicity, socio-economic status)
- Setting (including country and location)
- Outcomes (including barriers and facilitators, common themes)

3.1.5 Quality assessment

Although methods for assessing the quality of quantitative studies are well established (Higgins, 2008) the use of critical appraisal in systematic reviews of qualitative studies is more contentious. There are issues around quality assessment; both in terms of which quality criteria should be used and how that information should be applied to the review findings (Dixon-Woods et al., 2004, Sandelowski et al., 1997). However, the consensus appears to be that some form of quality assessment in a systematic review of qualitative studies is important (Campbell et al., 2003, Dixon-Woods et al., 2001, Noyes et al., 2008) as poor quality studies may distort findings and lead to difficulties in interpretation.

For this review we will employ a checklist used successfully by the team in previous work (Bunn et al., 2008). This is an adapted version of the framework for assessing quality in qualitative research designed by Spencer and colleagues (Spencer et al., 2003). Two reviewers will use the checklist independently and then meet to agree the final quality score. Discrepancies will be resolved by discussion or by consultation with a third author. As there is no consensus, or empirically tested method, for excluding studies from reviews on the basis of quality we will include all studies regardless of their quality. However, if possible, we will undertake sensitivity analyses to examine the relative contribution according to study quality.

3.1.6 Data analysis

A number of different approaches for the synthesis of qualitative studies have been proposed (Campbell et al., 2003, Sandelowski et al., 1997, Dixon-Woods et al., 2001, Harden et al., 2004). Marston and King in a systematic review of qualitative literature looking at factors that shape young people’s sexual behaviour (Marston and King, 2006) used comparative thematic analysis, to successfully synthesise a large number of studies. This approach uses well-established qualitative techniques and draws on existing literature around the synthesis of qualitative research (Campbell et al., 2003, Britten et al., 2002, Marston, 2004, Thomas and Harden, 2008). We will, therefore, synthesise the study findings using thematic analysis. In particular we will draw on the methods employed in two previous systematic reviews (Thomas and Harden, 2008, Marston and King, 2006).

Thematic analysis

Studies judged to have met the inclusion criteria will be independently reviewed and coded by two reviewers. This process of thematic synthesis will involve three stages; the coding of text, the development of descriptive themes and the generation of analytic themes (Thomas and Harden, 2008). Codes will represent themes that have emerged from the papers and will be refined by discussion and the use of constant comparison within and between codes to ensure that they accurately reflect the material. We will then identify associations between the different themes, grouping them into broad overall themes. These themes will then be used to answer our review questions (for example, identifying barriers and facilitators to early diagnosis and service provision) and where possible the themes will be used to go beyond the content of the original studies to construct larger narratives or general theories.

Defining what counts as ‘data’ and finding the key concepts in qualitative research is not always straightforward (Thomas and Harden, 2008). We will take the approach suggested by Thomas and Harden and take ‘data’ or study findings to be not just data in the form of quotations but to be all of the text labelled as ‘results’ or ‘findings’ in study reports (Thomas and Harden, 2008).

Results of the studies will be entered into NVivo software for qualitative data analysis. The use of software can facilitate the development of themes and allow reviewers to examine the contribution made to their findings by individual studies, groups of studies, or sub-populations within studies (Thomas and Harden, 2008).

Public involvement

In 2004 CRIPACC and HertNet (University of Hertfordshire) established a formal structure for public involvement. A Public Involvement in Research Group (PIRG) comprising membership across the PCTs, the PPIs, Children’s Fund, voluntary sector and existing lay members was established and works closely with INVOLVE. A member of this group (Heather Maggs a former dementia carer) has commented on the research proposal and will collaborate with the team for the duration of the project. She will be invited to join the project advisory group. As members of the advisory group they will have the opportunity to comment on the systematic review protocol and preliminary analyses, and be involved in interpreting and disseminating the review findings. The advisory group will meet three times over the course of the project but members will also have the opportunity to comment on outputs from the project such as preliminary and final reports.

References
