# Supplementary material for: Psychosocial Factors That Shape Patient and Carer Experiences of Dementia Diagnosis and Treatment: A Systematic Review of Qualitative Studies
Source: PLoS Med. 2012 Oct 30;9(10):e1001331. doi: 10.1371/journal.pmed.1001331 (PMC3484131; doi:10.1371/journal.pmed.1001331)
Supplement: Table S1 — Characteristics of included studies. (DOCX) [file pmed.1001331.s003.docx]

**Table S1: Characteristics of included studies**

| **Author*, year**  **Country** | **Perspective** | **Recruited from** | **Number participants**** | **Age PWD (mean & range where given)** | **Data collection methods** | **Methodology†** | **Analysis†** | **Principle experiences explored** | **Reliability** | **Usefulnes** |
| --- | --- | --- | --- | --- | --- | --- | --- | --- | --- | --- |
| Adams 1994  UK | Carers | PC | 6 |  | Diary analysis | E & GT | Case study | Emotional experience of informal caring | Low | Low |
| Adams 2006  US | Carers | MC | 20 |  | Interviews | P & GT | Thematic analysis | Caregivers subjective experience of early cognitive changes | Medium | High |
| Adamson 2001  UK | Carers | PC | 30 |  | Interviews | E&GT | Constant comparative analysis | Experiences of carers of S Asian & African Caribbean descent | Low | High |
| Bamford 2000  UK | PWD, Carers | Centre for older people with MH problems | 21 | 60-90+ | Focus Groups, informal group discussions | NS | Thematic analysis | Desired outcomes of community care | High | High |
| Banningh 2008  Netherlands | PW MCI | MC | 8 |  | Interviews | GT | NS | How PWMCI experience & cope with their cognitive decline | Medium | Medium |
| Beard 2004  US | PWD | Diagnostic centre | 13 | NS | Focus Groups, observation | P & GT | Constant comparative analysis | Impact of being diagnosed with early AD on identity construction | High | High |
| Beard 2008  US | PWD, PW MCI | Neurology clinic, psych clinic, Alz Soc | 40 | M = 71 (all over 65) | Interviews, Focus Group | E | Constant comparative analysis | Examine dynamics & context of focus groups for people diagnosed with dementia or MCI | High | High |
| Beard 2009  US | PW MCI or early stage dementia, carers | NS | 85 | 77% over 56 | Focus Groups | GT | Constant comparative analysis | Attitudes towards aging well from perspective of PWD & MCI | Medium | High |
| Beattie 2004  UK | PWD | Day centre for younger PWD | 14 | M 59 (R 41-66) | Interviews | GT | Comparative textual analysis | Experiences, care needs and views on services of younger PWD | Low | High |
| Benbow 2009  UK | Carers | Consumer grp (Royal College of Psychiatry) | 8 |  | Narratives | NS | Thematic analysis | Describe narratives arising from consumer group | Low | Medium |
| Blieszner 2007  US | People with MCI, Carers | MC | 146 |  | Interviews | GT | Thematic analysis | Application of the theory of ambiguous loss to couples with MCI | High | Medium |
| Boise 1999* (Connell 2004)  US | Carers | MC | 53 |  | Focus Groups, questionnaire | NS | Thematic analysis | Explore factors which delayed families in seeking diagnosis | Low | Low |
| Bowes 2003  UK | PWD, Carers | Via professionals & community grps | 8 | NS | Case studies | E | Thematic analysis | Experiences of S Asian families of service support | Low | High |
| Bruce 2000  Australia | Carers | PC | 24 |  | Interviews, questionnaire | NS | Thematic analysis | Access to community support & to determine potential barriers | Low | Low |
| Butcher 2001  US | Carers | Radio advertising & word of mouth | 103 |  | Unstructured interviews | P | Secondary phenomenological data analysis | Lived experience of caring | Medium | Medium |
| Byszewski 2007* (Aminzadeh 2007)  Canada | PWD, Carers | Geriatric day hospital (similar to MC) | 60 | M 82, R 71-92) | Interviews, focus group | NS | Constant comparative analysis | Process of dementia disclosure | High | High |
| Cahill 2004  Norway, Finland, Ireland and Lithuania | PWD | Health care professionals & service providers | 92 | M 76, R 54-97) | Interviews | NS | NS | Impact of assistive technology, quality of life | Low | Low |
| Cahill 2008  Ireland | PWD, Carers | MC | 56 | M 73, R 55-89) | Interviews | NS | Thematic analysis | Expectations & experiences of first appointment at memory clinic | Medium | Medium |
| Clare 2002a* (Clare 2002b, 2003a, 2003b)  UK | PWD, Carers | MC, & caseloads of clin psychol and Alz Soc worker | 24 | M 71, R 57-83 | Interviews | P | Interpretive phenomenological analysis | Ways PWD try to cope with changes | High | High |
| Clare 2004  UK | PWD, Carers | MC | 20 | R 52-83 | Recorded conversation between PWD & partner | Voice relational analysis | Voice relational analysis/conversational analysis | Ways couples use talk to construct an account of diagnosis | High | High |
| Clare 2005  UK | PWD | MC | 12 | M 71 | Interviews | P | IPA case study analysis | Early stage dementia using temporal framework | High | High |
| Corner 2006  UK | PWD, Carer | MC, Old age psych, Alz Soc, day centres | 3 | M 65 | Interviews | Case studies | Constant comparative analysis | Psycho-social implications for PWMCI | Low | Medium |
| Cotrell 1992  US | Carers | Alz Soc | 5 |  | Interviews | NS | Thematic analysis | Focus on emotional state/changes in early stage dementia | Low | Low |
| Davis 2011  USA | Carers | Recruited from RCT | 40 |  | Interviews | NS | Content analysis | Analysis of caregiver burden | Low | Medium |
| Deb 2007  UK | Carers | Old age psych | 24 |  | Interviews, Focus Groups | NS | Not stated | Carers perspectives of symptoms | Medium | Medium |
| Derksen 2006* (Derksen 2005, vernooij 2006)  Netherlands | PWD, Carers | MC | 36 | M 71 | Interviews | GT | Constant comparative analysis | Impact of disclosure of diagnosis on PWD & partner | High | High |
| De Witt 2010*  De Witt 2009  Canada | PWD | Health and social services | 8 | R 58-87 | Interviews | P | IPA | Meaning of living alone | High | Medium |
| Duggan 2008  UK | PWD, Carers | Old age psychiatry | 36 | R 71-84 | Interviews | GT | GT | Perspectives on outdoor environment | Medium | Medium |
| Duggleby 2009  Canada | Carers | Home-care co-ordinators | 17 |  | Interviews | GT | GT | Experience of hope | High | Medium |
| Frank 2006  US and UK | PWD (US&UK), People with MCI (US&UK), Carers(US) | Study Centre neurology/  geriatric psychiatrist | 67 | M – MCI 72, AD 77 | Focus Groups | NS | Content review | Identify key aspects of the impact of MCI & mild probable AD | Low | Low |
| Gillies 2000  UK | PWD | Health and social services | 20 | R 64-89 | Interviews | E | Line by line coding | Perspectives of PWD | Low | Medium |
| Gilmour 2003  UK | PWD, Carers | Sub-sample from previous study | 22 | M 83, R 74-93 | Interviews | NS | Template approach | Concepts of risk in PWD living alone | Low | Low |
| Gilmour 2005  New Zealand | PWD | Alz Soc | 9 | R 56-79 | Interviews | NS | Thematic analysis | Experience of living with memory loss | High | High |
| Hain 2010  US | Carers | Memory & wellness centre | 10 |  | Interviews | P | Descriptive phenomenological analysis | What matters most to carers | High | High |
| Harman 2006  UK | PWD | Alz soc | 9 | M 65, R 58-76 | Interviews | P | IPA | Self regulation model of illness behaviour | Medium | High |
| Harris 1999  US | PWD, Carers | Alz Soc | 32 | M 70, R 54-84 | Interviews | GT | GT | Concept of self in early stage dementia | High | High |
| Harris 2004* (Harris 2009)  US | PWD (US),people with MCI (US), Carers(UK) | Alz assoc & dementia advocacy grp | 38 | USA M 56, UK R 40-60 | Interviews, Focus Groups, online interview | GT | Based on Glaser & Strauss | Unique challenges of younger people with dementia & families | Medium | High |
| Hellstrom 2005  Sweden | PWD, Carers | MC | 40 | M 77, R 65-84 | Interviews | P | Constant comparative analysis | Experience of dementia over time, impact on relationships | High | High |
| Hinton 1999* (Fox 1999, Hicks 1999, Ortiz 1999)  US | Carers | Variety of health, social services, voluntary agencies | 7 |  | Interviews | NS | Narrative analysis | African American, Chinese American, Irish American, Latino caregivers | Medium | High |
| Hinton 2004  US | Carers | PC | 39 |  | Interviews | Narrative approach | Coding, developed typology of pathways to diagnosis | Pathways to diagnosis, help-seeking patterns & experiences across 3 ethnic groups | Medium | Medium |
| Holst 2003  Sweden | PWD | Old age psych | 11 | NS | Oral histories | Biographies | Biographical | Meaning of everyday life | Low | Medium |
| Howorth 2003  UK | PWD, Carers | Old age psych | 64 | NS | Interviews | NS | NS | Retention & loss of insight. | Medium | Medium |
| Hulko 2009  Canada | PWD, carers | Hospital outreach programme & Alz Soc | 16 | M 77, R 74-87 | Interviews, observation | GT | Constant comparative analysis | Relationship between experience & race, ethnicity, class & gender | Medium | High |
| Hutchings 2010, UK | PWD, Carers | MC | 23 | R 65-8R 46-865 | Interviews, focus groups | Thematic framework approach | Constant comparative analysis | The lived experience of CHEI users and the perceived impact of their treatment | High | High |
| Hutchinson 1997  US | PWD, Carers | Day care centre | 28 |  | Focus Groups, observation, field notes, autobiographical account | NS | Awareness context theory | Social interaction issues in early probable AD | Low | Medium |
| Jansson 2001  Sweden | PWD, Carers | MC | 16 | M 75, R 71-85 | Observation | GT | Content analysis | Patterns of elderly care giving | Medium | Low |
| Jutulla 2007* (Jolley 2009)  UK | Carers | From previous study | 15 |  | Interviews | NS | Thematic analysis | Asian carers experiences of services | Medium | High |
| Katsuno 2005  US | PWD | Day care centre | 23 | M 79, R 66-91 | Interviews | NS | Constant comparative analysis | Quality of life, personal experiences & reactions to negative view of dementia | Low | Medium |
| Keady 1999  UK | PWD | MC | 15 | R 72-84 | Interviews, field notes | GT | Constant comparative analysis | Coping behaviour, help seeking | Medium | High |
| Koppel 2007  UK | PWD, Carers (interviews not analysed) | MC | 6 | M 76 | Interviews | P | IPA | Experience of developing memory difficulties | Low | Medium |
| Kuo 2010  Taiwan | Carers of people with MCI | MC | 10 | PWMCI M 75, Carer M 50.8 | Interviews | GT | Constant comparative analysis | Experiences of caregiving process in Taiwanese families | High | Medium |
| Laakkonen 2008  Finland | Carers |  | 63 |  | Interviews | NS | Content analysis | Diagnosis, how information & support received | Low | Low |
| Langdon 2007  UK | PWD |  | 12 | M 79, R 66-87 | Interviews | P | IPA | Other people’s reactions to early stage dementia | Low | High |
| Lawrence 2008  UK | Carers | Community MH teams, and variety of carer services & voluntary organisations | 32 |  | Interviews | GT | Constant comparative analysis | Care giving attitudes & needs of carers from 3 ethnic groups | High | High |
| Lawrence 2010  UK | PWD | MC, MH teams, day centres, community MH programs | 30 | M – black 76, Asian 77, white 82 | Interviews, vignettes | GT | Constant comparative analysis | Subjective reality of living with dementia-from 3 ethnic groups | High | High |
| Leung 2011  Canada | PWD, carers | Geriatric clinic, Alz Society | 13 (6 PWD, 7 carers) | PWD 3 in 70’s, one in 60’s and one in 50’s | Interviews | P | Inductive thematic analysis | Experiences of problem recognition and obtaining a diagnosis | High | High |
| Lingler 2006  US | People with MCI | MC | 12 |  | Interviews | GT | GT- coding & thematic analysis | Living with diagnosis of MCI | High | High |
| Livingston 2010  UK | Carers | PC, MC | 89 |  | Interviews, Focus Groups | GT | Thematic analysis | Common difficult decisions made by carers | High | High |
| Lu 2009  US | Carers | MC | 10 |  | Interview | P | Thematic analysis | Commonalities of lived experience | Medium | High |
| MacQuarrie 2005  Canada | PWD, Carers | MC, voluntary org | 26 | M 76.5, R 60-89 | Interview | Hermeneutics | Constant comparative analysis | Experiences of PWD | High | High |
| MacRae 2010  Canada | PWD | MC | 9 | R 60-85 | Interview | P | Constant comparative analysis | Impact of AD on self identity | Low | High |
| Mahoney 2005* (Cloutterbuck 2003, Neary 2005)  US | Carers | Alz assoc | 22 |  | Interviews, Focus Groups | NS | Content analysis & meta-synthesis | Cross cultural similarities & differences of 3 ethnic grps towards onset & diagnosis | Medium | Medium |
| Manthorpe 2011 | PWD/MCI, carers | MC | 53 | 18 aged 65-79, 5 over 80, 3 under 65 | Interviews | Drew upon GT methods | Constant comparative analysis | Experiences, expectations and service needs of people with memory problems and their carer | High | High |
| Mason 2005  UK | People with MCI | Day centre older adults | 11 | M 79, R 72-86 | Interviews, analysis of video footage | P | IPA, behavioural interactional codes | Mutual support processes | Low | Medium |
| Menne 2002  US | PWD | Neurology & Alz dis Centre support grp | 6 | M 71.7, R 57-88 | Interviews |  | Strauss, A (1987), Qualitative analysis | Day to day activities | Medium | Medium |
| Mok 2007  Hong Kong | PWD | Community rehabilitation network | 15 | R 56-80 | Interviews, observation | P | Line by line coding | Living & coping amongst older Chinese people | Medium | High |
| Monzin-Cook 2006  UK | PWD, Carers | MC | 96 | M 76.7, R 66-87 | Interviews | P | IPA | Understanding about dementia before attending memory clinic | High | High |
| Moreland 2001,  UK | Carers | Housing associations, churches, voluntary grps | 20 |  | Interviews | NS | Thematic analysis | Service experiences of BME groups | Low | High |
| Moreland 2003  UK | Carers | Voluntary grps, churches | 19 |  | Interviews | NS | Thematic analysis | Service experiences of BME groups | Low | High |
| Mukadam 2011 | Carers | PC | 18 |  | Interviews | GT | Coding | Effect of culture and ethnicity on help seeking for dementia symptoms | Low | High |
| Murray 1999  14 EU countries | Carers (20 from each country) | MC | 280 |  | Interviews | GT | Content analysis | Comparison of subjective experience across Europe | Low | Medium |
| Neufeld 2003  Canada | Carers | Adverts in community newsletters, health care agencies | 8 |  | Interviews | Symbolic interactionalism | Thematic content analysis | Non-support for care givers | Low | High |
| Nygard 1998  Sweden | PWD | In-pt diagnostic unit | 2 | Mid 50s | Interviews | P | IPA | Detailed longitudinal case studies | High | High |
| O’Connor 1999 | Carers | Community support service organisations | 14 |  | Interviews | Discourse analysis/case study | Discourse analysis | Interface between living with memory impaired spouse & use of formal support services | High | High |
| Ostwald 2002  US | PWD | Participants from previous study | 56 | M 77.6, R 47-97 | Interviews | NS | Thematic analysis | Losses, feelings, coping | Low | Medium |
| Pearce 2002  UK | PWD, Carers | MC | 40 | M 75 | Interviews | P | IPA | How diagnosed men deal with onset of disease | Medium | High |
| Perry 2002 | Carers | NS | 38 |  | Interviews | NS | Constant comparative analysis | Strategies used by spouses to preserve personhood of partner | Low | Medium |
| Phinney 1998  Canada | PWD, Carers | research centre | 10 | R 75-89 | Interviews, observation | NS | Thematic analysis | Living with AD & what it means | Medium | High |
| Phinney 2006* (Phinney 2002, Phinney 2003, Phinney 2007)  Canada | PWD, Carers | Research centre & support grp | 16 | R 64-88 | Interviews | P | Interpretive phenomenological analysis | How family members support meaningful activity for PWD | High | High |
| Pollitt 1989  UK | Carers | PC | 34 |  | Interviews | NS | NS | Perceptions & responses to deterioration in intellectual function | Low | High |
| Post 2001  USA | PWD, carers | Alz assoc | 17 | M 71 | Focus Groups | NS | Thematic analysis | Views on impact of cognition-enhancing medication on quality of life | Low | Medium |
| Pratt 2003* (Pratt 2001)  UK | PWD | PC, MC | 24 | R 44-78 (11 under 65 and 13 over) | Interviews | NS | Thematic analysis | Impact of social context | High | High |
| Preston 2007  UK | PWD | MC | 12 | M 71, R 58-81 | Interviews | P | IPA | Coping strategies | Medium | High |
| Quinn 2008  UK | Carers | MC | 34 |  | Interviews | P | IPA | Daily experience & accessing support | Medium | High |
| Roberto 2011  USA | People with MCI and their carers | MC | 168 (56 PWMCI, 112 carers) | PWMCI M 76.5, primary carer M 66.8 | Interviews | GT | Content analysis | Impact of memory changes on family dynamics | High | High |
| Robinson 1997* (Robinson 1998)  Sweden | People with MCI | MC | 8 |  | Interviews | P | Hermeneutical /Phenomenology approach | Pre-diagnosis | Medium | High |
| Robinson 2005  UK | PWD, Carers | MC | 18 | M 77, R 65-85 | Interviews | P | Interpretive phenomenological analysis | Psychological reactions to diagnosis in couples | High | High |
| Samuelsson 2001  Sweden | Carers | Group housing | 8 |  | Interviews | NS | Content analysis | Care giving for elderly | Low | Medium |
| Seabrooke 2004  UK | Carers | Voluntary grps | 7 |  | Interviews, Focus Groups | GT | Content analysis | Service needs of Asian older people | Medium | High |
| Shaji 2003  India | Carers | NS | 17 |  | Interviews | NS | Constant comparative analysis | Care arrangements, attitudes, sources of strain | Medium | Low |
| Smith 2001  Canada | PWD, people with MCI, Carers | MC | 40 | Median, AD 85, no AD 49 | Interviews | NS | Inductive reasoning | Lay understanding of diagnostic information | Low | Low |
| Steeman 2007  Belgium | PWD, Carers | MC | 40 | R 69-91 | Interviews | GT | Grounded theory & narrative research | What it is like to live with early stage dementia | High | High |
| Sterritt 1998  US | Carers | NS | 9 |  | Interviews, open-ended questionnaires | NS | Thematic analysis | Cultural attitudes, beliefs & values of African American carers | Low | Low |
| Svanstrom 2004  Sweden | PWD, Carers | PC, MC | 10 | R 73-80 | Interviews | P | Phenomenological | Lived experience of dementia in couples | Medium | Medium |
| Teel 2003  US | Carers | Alz assoc | 14 |  | Interviews | NS | Constant comparative analysis | Barriers to recognition & treatment | Medium | High |
| Todres 2006* (Galvin 2005)  UK | Carer | Participant approached researcher | 1 |  | Life-world descriptions | P | Phenomenological narrative analysis | Carer experience post diagnosis | Medium | High |
| Van Dijkhuizen 2006  UK | PWD, Carers (not reported) | PC | 18 | M 78, R 70-86 | Interviews | P | IPA | Gender differences in coping strategies & appraisal | Medium | High |
| Vellone 2002  Italy | Carers | Medical centre | 26 |  | Interviews | P | Phenomenology | Italian family members experiences | Low | Medium |
| Watkins 2006  UK | PWD | Psycho-therapy group | 1 | M 76 | Focus Groups | NS | Stiles et al | Changes occurring during psychotherapy group | Medium | Medium |
| Werezak 2002  Canada | PWD | Not clear | 6 | R 61-79 | Interviews | GT | Constant comparative analysis | Learning to live with memory loss | Medium | High |
| Westius 2009  Sweden | PWD | MC, day care centre | 21 | R 73-89 | Interviews | P | Hermeneutical phenomenology | Life story | Low | Low |
| Wolverson 2010  UK | PWD | MC | 10 | M 81.8, R72-87 | Interviews | P | IPA | Subjective experience of hope | High | Medium |

* = primary reference (associated references in brackets)

** PWD/MCI and carers only, † as reported by study authors

PC = primary care, MC = memory clinic, MH = mental health

GT = Grounded theory, E = ethnography, P= phenomenology, NS = not stated
