# Supplementary material for: Psychosocial Factors That Shape Patient and Carer Experiences of Dementia Diagnosis and Treatment: A Systematic Review of Qualitative Studies
Source: PLoS Med. 2012 Oct 30;9(10):e1001331. doi: 10.1371/journal.pmed.1001331 (PMC3484131; doi:10.1371/journal.pmed.1001331)
Supplement: Table S2 — Themes and supporting evidence. This table shows the themes that arose from our analysis and the evidence to support them. (DOCX) [file pmed.1001331.s004.docx]

**Table S2: Themes and supporting evidence**

| **Theme 1 – Pathways through diagnosis** | |
| --- | --- |
| **Subthemes** | **Supporting evidence (+ indicates high reliability, ~ medium and – low)** |
| **Barriers & facilitators of earlier diagnosis** | - Trigger event/life event/tipping point potentially challenges pattern of normalisation of symptoms of dementia as old age (but carers/pwd may still may not be able to recall when symptoms first began) Beard 2004+,Beard 2008+, Hutchinson 1997, Adamson 2001, Bowes 2003-, Butcher 2001~, Clare 2004+, Deb 2007~, Hutchinson 1997-, Lu 2009~, 57Mahoney 2005~, Moreland 2007, Quinn 2008~, Seabrooke 2004~, Banningh 2008~, Blieszner 2007+,Corner 2006- - Medical professional misdiagnose/attribute symptoms to old age (Mahoney 2005~, Seabrooke 2004~), GP misdiagnosis (Livingstone 2010 +, Beattie 2004-, Juttlla2007-) - Common that the PWD doesn't recognise that something is wrong, however diagnosis can validate carer suspicions (Adamson 2001-, Blieszner 2007+, Hutchinson 1997-, Livingston 2010+, Werezak 2002~) (Boise 1999-, Byzewski 2007+, Derksen 2006+, Robinson 2005+), carer may have to convince loved one there is something wrong with them (Livingston 2010+), may not be joint act between carer & PWD (Manthorpe 2011+). - Some carers reported there was a delay in diagnosis (Benbow 2009-, Livingston 2010+ , Seabrooke 2004~, Holst 2004-), waiting between appointments time of uncertainty and worry (Manthorpe 2011+) - Patients and carers have little understanding of diagnosis process and service pathways (Manthorpe 2011+) - PWD could convince the doctor that there was nothing wrong (Harris 2004~, Livingston 2010+). - Carers from ethnic minority backgrounds could recognise the term dementia but it was not widely known about (Adamson 2001-,Shaji 2003~). No equivalent word for dementia in Asian languages (Seabrooke 2004~). - Experience of memory clinic polarised in positive and negative experiences (Koppel 2007-). Similarities across countries in terms of processes of diagnosis (Bowes 2003-). - People could not always articulate the fact that terms such as AD or dementia were used or they found the term frightening (Byzsewzski 2007+,Pratt 2001+, dementia/AD is a frightening term (Gilmour 2003-, Lingler 2006+, Aminzadeh 2007). - Interpreters are sometimes inaccurate (Juttlla 2007-). - Confidentiality obstacles (Livingston 2010+, Juttlla 2007-), difficult to ask questions or talk about PWD in front of them (Manthorpe 2011+) - Belief it better to ‘catch it early’ (Manthorpe 2011+) - May seek help in order to have anxiety quelled (e.g.not AD) (Manthorpe 2011) |
| **Challenges to identity** | - Emotional reactions ranged from shock, anger, grief, and confirmation of suspicions (Byszewski 2007+, Corner 2006-, Pratt 2001+, Werezak 2002, Harman 2006~, Pratt 2001+, Clare 2004+, Pratt 2001+, Beattie 2004-.), positive coping responses (Byszewski 2007+, Werezak 2002~, Manthorpe 2011+), not being in control (Phinney 2006+),. - Internal dialogue- Coming to terms with diagnosis. Carers often helped care recipients to maintain a sense of former self (Sterritt 1998 -) - Carers had to come to terms with partners losses and their own shifts in role and identity (Lawrence 2008+) - PWMCI persisted in maintaining hobbies (Blieszner 2007+) - Loss of skills and former identity (MacRae 2010-, Hutchinson 1997-, Steeman 2007+.) - It was sometimes impossible for carers to enable PWD to maintain certain aspects that were strongly associated with their sense of self e.g. driving (Livingston 2010+, Byszewski 2008+) - Frustration- loss of skills often linked to identity and communication (Langdon 2007-, Harris 2004~, Hain 2010+). Loss of ability to drive- Beard 2008+, Gilmour 2003+, 2005+, Howarth 2003~, MacQuarrie 2005+, Pratt 2001+, Harris 1999~.) - Sadness-negative impact on self-esteem (possible depression) (Langdon 2007-, Bamford 2000+, Clare 2004+, Phinney 2006+, Gillies 2000-, Koppel 2007-, Deb 2007~, Derksen 2006+, Ostwald 2002-, Banningh 2008~). - Eventual acceptance (Beard 2009+, Byszewski 2007+, Livingston 2010+, MacRae 2010-, Mok 2007~,Werezak 2002~, Pearce 2002~, Preston 2007~, Van Dijkhuizen 2006 ~, Manthorpe 2011) - PWD may value the outdoor environment but carer becomes anxious about their ability to navigate in their locality (Hulko 2009~) |
| **Changes to roles & relationships partner and nuclear family** | - Key carer and recipient of care have to adjust to progressively unequal relationship (Adams 2006~, Harris 1999~, Harris 2004~, Derksen 2006+, Monzin -Cook 2006+, Quinn 2008~, Svanstrom 2004~, Todres 2006~, O’Connor 1999) - Carers often focused on PWD abilities rather than mistakes or lack of recall Hellstrom 2007+, Phinney 2006+), carer compensates for PWD but tries to prevent the PWD from noticing (- O’Connor 1999). - Much of the focus was on couples and many emphasised working together as a team with a high degree of mutuality (Benbow 2009-, Clare 2002+, Clare 2004+, Livingston 2010+, MacRae 2010-, Pollitt 1989-, Manthorpe 2011+) - Some couples adapted well to the changes they were presented with including recognition of separate needs of partner (Butcher 2001~, Hellstrom 2007+, Todres 2006~) - PWD become involved in an active process of self-preservation (Beard 2004+, Beard 2008+, Holst 2003). - PWD living alone can miss lack of contact from family and may be dealing with the distress caused by loneliness as well as coping with dementia (Cahill 2004-, Van Dijkhuizen 2006~) - Misconceptions/prejudices amongst wider family (Katsuno 2005-, Moreland 2007, Seabrooke 2004~) - PWD and carer interpret things differently therefore meaning is negotiated (Pearce 2002, Hellstrom 2007 +) - Dementia often compromises ability to communicate or willingness to do so (Phinney 2006+, Deb 2007 ~, Cotrell 1992 –, Hellstrom 2007+, Quinn 2008~), PWD may become less engaged (Swanstrom 2004~, Holst 2003-) - Guilt- PWD guilt over carer burden (Cahill 2004-, Gillies 2000-, Mok 2007~, Ostwald 2002-) - Carers had to adapt to increasing responsibility and the lack of prospects for sharing or relinquishing that role (Butcher 2001~) - Carers health adversely affected as a result of strain of providing constant care- emotionally drained also (Bruce 2000-, Butcher 2001~, Hain 2010+, Laakkonen 2008-, Samuelsson 2001-, i.e. emotional and physical symptoms including frustration and sleep deprivation (Bruce 2000-) - Persuading partner to stop driving (Derksen 2006+, Livingston 2010+, Byszewski 2007+, MacQuarrie 2005-, Menne 2002~, Pratt 2001+, Steeman 2007+). |
| **Changes to roles & relationships: wider social networks** | - PWD withdrawing from social network- weakened relationships with family or social network (Harman 2006~, Ostwald 2002-, Deb 2007~, Duggan 2008~, Holst 2003, Manthorpe 2011). - Non intentional isolation from wider social networks occurred as did intentional distancing (Bowes 2003-, Katsuno 2005-, Koppel 2007-, Neufeld 2003-, Shaji 2003~, Svanstrom 2004~, Teel 2003~, Todres 2006~) - Decisions over whether to disclose diagnosis to wider circles was often determined by the carer/PWD ability to normalise the situation in doing so (Hutchinson1997, MacQuarrie 2005-, MacRae 2010+, Mason 2005). - PWD may become extremely mindful of who may be aware of their diagnosis (Langdon 2007-) - Other forms of non-communication include withholding diagnosis or increasingly severe memory problems from others by maintaining a façade (Cottrell 1992-) |
| **Theme 2: Resolving conflicts to accommodating a diagnosis** | |
| **Acceptability of support (both formal and informal)** | - Caring assessed as a private responsibility; an assumption exists that spouses will provide the best care therefore carers set a precedent for believing other care services to be inferior. (O’Connor 1999) - Carers avoided services by discursively positioning them as potentially ‘patronizing’ to their partner. E.g. clinicians who may talk to their partner as if they are a ‘non-person’ Perry 2002. - Support needs sometimes considered unavailable or unacceptable to BME communities (Sterritt 1998-) - Caregiver acted as a bridge between the public and private world of the PWD (Benbow 2009-) - carers from BME communities felt they had not been helped to become knowledgeable (Moreland 2007) - In wider social /familial relationships support and non-support alternated over time(Bowes 2003-, Neufeld 2003-, Teel 2003~) - Those who seek help earlier may have different expectations than those who seek help later (Manthorpe 2011). - Traditional and non-traditional carer ideologies altered the carer approach and interaction with services. Traditional caregiver ideology (Lawrence 2008+, Ostwald 2002 -, Sterritt 1998 -, Vellone 2002 -). Others expressed non-traditional caregiver ideology (Lawrence 2008+) - Carers torn between protecting and advocating for the PWD and facilitating some level of independence (Protecting & supervision - Murray 1999-, Quinn 2008~, Hulko 2009~). Facilitating independence - Howorth 2003, Jasson 2001, Livingston 2010,) meaningful activity important Bamford 2000.) |
| **Living in present and dealing with anxiety about future** | - Uncertainty over future and prognosis (Howarth 2003~, Koppel 2007-, Ostwald 2002-, Manthorpe 2011) - Many thought about potentially having PWD put into a care home (Gilmour 2005+, Monzin-Cook 2006+, Steeman 2007+, Vellone 2002 -, Samuelsson 2001 -), carers worried will be unable to cope (Manthorpe 2011) - PWD worried about becoming a burden (Cahill 2004 -, Gillies 2000 - ) - Carers worried that if something else happened to the PWD they would not be able to cope on their own (Phinney 2006+, Livingston 2010+, Oswald 2002 -) - People focused on day to day living however, living with uncertainties about the future can make it difficult for PWD to maintain sense of self (Duggleby 2009+, Gilmour 2005+, MacRae 2010 -, Mok 2007+, Manthorpe 2011+) - Hopes for the future were not to see their loved ones' condition get worse and to hope for results from the medication (Vellone 2002 -) - PWMCI- ambiguity about the future prevents people from adjusting to their loss (Blieszner 2007+) - Fear that likely to face battle with services (Manthorpe 2011+) - Fear of future and ability to cope without more intensive care (Phinney , 2006), fear of future (Gilmour 2005+, MacQuarrie 2005+, Hutchinson 1997-, Ostwald 2002-, Fear & frustration (Adams 2006~, Holst 2003-) - PWD aware of carer's constant worry & vigilance for signs of deterioration (Monzin-Cook 2006, Robinson 2005) - Carers are motivated to continue in their role, but may lose a sense of hope for improvement in circumstances (Duggleby 2009+) |
| **Usefulness or harmfulness of knowledge** | - Knowledge could be a burden for a carer (Byszewski 2007+) - Split between carers who actively seeking information and those who did not want to face the future (Adams 2006~, *Fear of future-* MacQuarrie 2005+, Gilmour 2005+) - PWD experience confusion about symptoms (sometimes described as denial)( Pollitt 1989-,Corner 2006-) - Lack of understanding of problems associated with dementia (Juttlla 2007-), unreceptive to further information (Keady 1999~) - Alternative Cultural /religious explanations (Seabrooke 2004~) - PWD reluctant to disclose their diagnosis due to their continued perception of the term AD and what they thought it would mean to others- misconceptions were felt to be prevalent (Langdon 2007-) - Timing of information important (Manthorpe 2011+), may be overwhelmed by information in early stages - Learning about dementia could help people to cope (MacQuarrie 2005+) - Perceived dementia and AD in terms of extreme scenarios (Manthorpe 2011+) - Carers & PWD could continue to consider memory loss as insignificant after diagnosis (Corner 2006-, Moreland 2007, Shaji 2003~, Manthorpe 2011, Pearce 2002) |
| **Theme 3: living with dementia** | |
| **Strategies to minimise the impact of dementia** | - Minimization of losses and normalising adaptation was a feature of the early stages of dementia especially amongst couples. Oscillation between recognising losses and developing coping strategies (Robinson 2005+) minimising losses, (Gillies 2000-, Howarth 2003~, MacQuarrie2005+, Swanstrom 2004~, Clare 2002+, Clare 2004+, Practical strategies, Gillies 2000-, MacQuarrie 2005+, Perry 2002-, Manthorpe 2011+) - Variety of positive and negative mind-sets, often constructed jointly by PWD & carer (positive Clare 2004+, Hellstrom 2007+, Butcher 2001 ~, Duggleby 2009+, Beard 2004+, Manthorpe 2011+, negative Laakkonen 2008 -, Gilmour 2005+) - Negative mind-sets could take hold e.g. hopelessness and loneliness (Laakkonen 2008 -, Phinney 2006+) - There may be a process of taking stock and dealing with the diagnosis before mind-set can be more positively re-framed later on (Keady 1999~) - Attitudes include a conscious rejection of strategies in adopting a pragmatic mind-set (MacQuarrie 2005+, Lu 2009 ~, Hain 2010+) - Everyday practical strategies/prompts (Corner 2006-, Gillies 2000-, MaQuarrie 2005+, Duggan 2008~, Manthorpe 2011+, Pearce 2002) - Social strategies - relying on family support, being helpful to others and isolating themselves from others (Ostwald 2002 -, Manthorpe 2011+) - Carer strategies and concealment of emotions e.g. becoming an emotional cheerleader (Adams 2006~, Bruce 2000 -, Butcher 2001~) - Humour (Beard 2004+, Beard 2008+, Menne 2002~) - Staying active (Beard 2009), Carrying on as normal and maintaining independence (Manthorpe 2011) - Actively finding meaning and joy (Butcher 2001~, Cahill 2004-,Pratt 2001+) - Faith/religion (Butcher 2001~, Sterritt 1998-) - Managing identity was a way of managing dementia (Preston 2007~). - A strategy may be to relinquish the things that you cannot continue (Harman 2006~), setting lower limits (Pearce 2002) - Carer’s resilience is representative of the couple’s ability to cope (Clare 2004+) |
| **Support from professionals and agencies** | **Support from GP and memory clinics**   - After going to the GP patients and carers said they were signposted /referred to other services/forms of support (Jutulla 2007-), GP usually first point of contact (Manthorpe 2011+) - Family members also wanted separate time with physicians to discuss diagnosis and symptoms (Teel 3003 ~,Pearce 2002~) - PWMCI/D who attended memory clinics and found the socialisation with people with dementia shocking and frightening (Corner 2006-, Katsuno 2005-, Mason 2005-, Manthorpe 2011+) - Delays in referral contributed to carer difficulties (Bruce 2000-, Hutchinson 1997-, Livingston 2010+.)   **Post-diagnosis support**   - Support and information from Alzheimer's society (Quinn 2008~, Manthorpe 2011+,Pearce 2002 ~) - It is important for HPs to provide information about services available and encourage carers to access these services early in the care trajectory (Hain 2010 +) - Expectations of more follow-up care (Laakonnen 2008-, Werezak 2002 ~, Hain 2010+) - Carer strain/burden prevalent and respite is required (especially as result of constant supervision necessary) (Benbow 2009-, Bowes 2003-, Butcher 2001~, Duggleby 2008+, Moreland 2007, Phinney 2006+, Shalji 2003~, Svanstrom 2004~, Vellone 2002-, Todres 2006~) - Community care- choice important also regular morning and evening calls, nutritious meals (Bamford 2000-) - People living alone- the locality was extremely important in managing risk (Gilmour 2003-) - The specialist NHS projects and the voluntary sector projects appeared to be working at full stretch (Bowes 2003-) - Importance of being able to use info to talk, understand and listen (Lu 2009~). - Cultural competence was regarded as important and carers wanted specialist services or specialist knowledge integrated into services where it was absent (Seabrooke 2004~) - Following the assessment the carers felt relief that there would now be access to services for carer and PWD (Juttlla 2007-). - Timing of referral from the GP to community support was considered important (Bruce 2000-, Pearce 2002~, Laakonnen 2008-) - Services perceived as being inappropriate for younger people (Beattie 2004-) - Confidentiality issues acted as obstacles for carers in obtaining information from health professionals (Seabrooke 2004~, Livingstone 2010 +) - Carers unsure if they are doing the right things and they question reactions to behaviours (e.g. depression Howarth 2003~, e.g. hallucinations –Samuelsson 2001-, PWMCI personality changes Lu 2009~)   **Medication**   - Some had renewed hope as a consequence of taking medication and new and emerging drug therapies were considered successful (Clare 2002+, Pratt 2001+, Todres 2006~, Post 2001-) Attitudes include ambiguity over benefits; whilst others (PWD) noticed small improvements and others thought medication would ensure they maintained a particular level of functioning and got no worse. Side effects were taken into account in the way in which patients weighed up the benefits of the medication e.g. sleep deprivation. (Hutchings 2010+). Expectation that medication would be available (Manthorpe 2011+)   **Peer support**   - Support groups gave these couples facing the similar types of losses the opportunity to speak about it (Werezak 2002~, Wolverson 2010+, Beattie 2004-, Hutchinson 1997-, Manthorpe 2011+) |
