# Supplementary material for: Psychosocial Factors That Shape Patient and Carer Experiences of Dementia Diagnosis and Treatment: A Systematic Review of Qualitative Studies
Source: PLoS Med. 2012 Oct 30;9(10):e1001331. doi: 10.1371/journal.pmed.1001331 (PMC3484131; doi:10.1371/journal.pmed.1001331)
Supplement: Table S3 — Examples of quotations illustrating themes and author interpretations of findings. This table provides examples of quotes supporting the themes from our analysis and gives examples of the ways authors of our included studies interpreted their findings. (DOCX) [file pmed.1001331.s005.docx]

**Table S3: Examples of quotations illustrating themes and authors interpretation of findings**

| **Themes** | **Quotations from participants** | **Study authors interpretation of findings** |
| --- | --- | --- |
| **1. Pathways through diagnosis** | | |
| **Barriers and facilitators of earlier diagnosis** | It never occurred to me that my mom had AD even though I knew something was wrong. . . . People in Chinatown told me that my mom was getting old and became Lao Hu Tu [stupid /forgetful in old age] (Mahoney 2005)  I say we all get forgetful. It’s something that just comes along (Quinn 2008) | The care-givers tended to normalize the changes in the care-recipients,  believing they were just part of the normal aging process (Quinn 2008) |
|  | erm, because I don’t think my memory really is any worse than friends. All our friends tend to be round … well, close friends tend to be round about our age and we’re all complaining about the same things, you know, you ask questions like have you seen my keys, do you know where my purse is … and, you know, that sort of memory … and I think that is mostly lack of concentration ... (Manthorpe 2011) | Recognising something wrong and seeking help slow process of internal recognition and acknowledgement, external acknowledgement within families and relationships and finally a slow journey through health services (Manthorpe 2011) |
|  | Well, I’ve been playing [cards] with a foursome for a number of years. . . So one day when I was playing a hand, all of a sudden I said to them, ‘I don’t remember what trump is’ and that’s when I started thinking, ‘I wonder why would I forget that, that’s basic? (Beard 2004)  He (dad) went into hospital and it was then because mum was on her own that we realised (Manthorpe 2011) | Typically, a specific episode, or turning point eventually led participants to seek medical attention for their forgetfulness and subsequently be diagnosed (Beard 2004) |
|  | It took me about eighteen months to get [the doctor] to . . . give her a test . . . later . . . my daughter . . . found the gas turned on and it’s not alight, so . . . when I left for work I had to turn the gas off, then I really pushed the doctor. (Livingstone 2011)  I first took her to the doctor, and we told about what was going on with the forgetfulness. . . . Then we went to the second, then back to the first, and a neurologist, too, but he never gave us a diagnosis or anything, and then we went to a psychiatrist the last time. We were just searching for something. Did she have Alzheimer’s, or was it something else? (Teel 2003) | Much energy and effort was expended in getting the elder there, usually amid protests, only to be disappointed at the encounter. Some physicians dismissed reported symptoms as simply old age; others believed the elders who denied problems, and they reported that these individuals were just fine. (Mahoney 2005) |
|  | On the phone the people would say ‘well we’d have to speak to your mother first to get permission to talk about her issues’ because you know they couldn’t say anything to me. . . I have to get my mother’s permission to represent her. (Livingstone 2011) | Confidentiality may impede carers receiving information (Livingstone 2011) |
|  | I wanted to be validated. I no longer wanted to be viewed by my family as the crazy one (Boise 1999)  we needed the assessment to find out what was wrong with her and at least we know what it is and we can work from there. It confirmed our suspicions (Byszewski 2007) | Sometimes carers were relieved or felt validated by a diagnosis (Boise 1999) |
| **Challenges to identity** | I’m still the same person I’ve always been. It’s just  that now I’m me with Alzheimer’s (Beard 2008).  . . . and when I go there, I go to be a woman. It’s the women’s group. It’s not like when I go to the memory club to be, um, well to be with other people with Alzheimer’s…I’m being most conscious of the woman bit with the women and the Alzheimer’s bit at the club (Preston 2007). | Participants forced to strategically navigate their identity or accept a compromised one (Beard 2008).  Tension could arise between acknowledging the nature and implications of dementia and yet having a sense of being the same person (Harman 2006).  People attempt to maintain an image, both publicly and personally, of a self that is ultimately in danger of being shattered. These people try to resist being assigned the ‘master status’ of a ‘demented’ person (Beard 2004) |
|  | ..... it was almost like a death. (Byszewski 2007)  Well, I was just devastated. (Werezak 2002) | The process of oscillation couples described in making sense and adjusting to loss following receipt of the diagnosis appeared to be similar to the process of adjustment outlined in dual-process models of grief. (Robinson 2005) |
|  | Veronica said we must tell them at the club that I have dementia. But I won’t tell them anything.. I think they may yap, yap, yap with everybody that I have dementia (Langdon 2007) | Some employed information management strategies to protect self-image and identity (MacRae 2010) |
| **Changes to roles and relationships** | We always hope to be together. That is what we say every night, ’I can’t manage without you’ he tells me. He is incredibly thankful for whatever I do. I don’t want him to feel like a burden for me. Instead I always try to cover up (some of my caring) (Hellstrom2007). | Losing their natural roles in the home further contributes to those with dementia growing more dependent on their spouses and others (Svanstrom 2004). |
|  | I think the disease itself is enough problem but the constrictions that they [family members, doctors, etc.] place around you: you can’t do this, you can’t do that; you can’t drive. You end up being extremely frustrated (Beard 2008). | They experience a range of emotional reactions as they struggle to maintain their sense of personal identity, emotions such as frustration, embarrassment/humiliation, anger, despondency, and uselessness (Harris 1999). |
|  | Just kind of stressed out. So it’s the constant care giving. You do not ever forget about it. You may be doing something else to try to enjoy yourself, but it is always in the back of your mind (Butcher 2001) | Caregivers were engaged deeply in the day-to-day work of caregiving. (Butcher 2001) |
|  | You can’t just do it yourself. You have to ask. So you have to adjust your schedule to someone else’s. I guess the best word for it is that it is somewhat humiliating to be in that position when you’re used to running your own life (Beard 2008). | Several families were learning how to carefully structure activities, reducing the demands of the involvement in such a way that the person with dementia was able to cope successfully (Phinney 2006).  No matter how positively persons with AD define the situation themselves, the success of their efforts to manage identity will depend on the support and cooperation of others. (MacRae 2010) |
|  | My family members’ relationships [with me] changed as soon as they found out that I was ‘‘no longer competent.’’ The things that I say seem to be a lot more subject to question than they used to be. It’s as if I can’t possibly know anything anymore. At least that’s the way I feel (Beard 2008). | So he or she enters a changing process of ‘‘negotiation’’ with the loved one, in a sense, ‘‘asking for permission’’ to take over in areas that may require assistance (Todres 2006).  Other social relationships may lose their ease and there may be a social renunciation that throws the carer and the loved one together more intensely in a new way that requires adjustment. (Todres 2006) |
| **Changes to wider social networks** | In my whole life I have been active in many choirs. Now, when I no longer remember the songs, I have decided to give up that activity . . . not funny to do things bad when you have been a “master” (Holst 2003). | Negative stereotypes about the meaning of a diagnosis may lead people with dementia to withdraw from others to maintain secrecy as well as contributing to others withdrawing social support. (Robinson 2005) |
|  | I have always found it easy to get on with people, I can’t do so anymore and it hurts . . . . (Holst 2003) | PWD felt they had lost the ability to reach out to others and were therefore no longer acknowledged as a person (Holst 2003) |
|  | They [friends] don’t know what to say. And when they do come and John seems disinterested or he’s not talking or he’s talking in a way that they can’t understand, they just hesitate to come back (Neufeld 2003) | Others want to distance themselves from the realities of dementia (Todres 2006). |
| **Theme 2: Resolving conflicts** | | |
| **Acceptance of need for services** | He refused all help. He wouldn’t let anyone come into the house, no form of carer (Livingstone 2011).  I just try to keep an eye on when is the time that I know I can’t handle or take it. You know it will, of course, get worse, that I know (Betts Adams 2006). | Feelings of inadequacy as a caregiver, including guilt about their angry feelings towards the person with memory loss, appear to make it more difficult for people to seek support from others. (Betts Adams 2006) |
|  | If it [going to a nursing home] happened tomorrow, I wouldn’t feel bad about it, you know, I mean I’ve had a really good life. . . One of the things you do is you sort of review your life. . . I mean, I’ve just had a tremendous life (MacRae 2010) | The life review can be a means by which illness is integrated into the self and identity renegotiated (MacRae 2010). |
|  | No. I don’t have any trouble with my memory. I don’t have any trouble with my memory! People, other people might have trouble with my memory, but I don’t have any trouble . . . As far as I’m concerned, Alzheimer’s is not bothering me at all! (MacQuarrie 2005) | Resistance to knowing – some participants did not construct AD as particularly relevant to their experience (MacQuarrie 2005)  Did not see it affecting their lives in a negative way, although could be lack of insight should also be seen in context of life lived (Manthorpe 2011) |
| **Living in present and dealing with anxiety about future** | I’ve kind of refused to look at the future because it’s nothing I have control over. All I can do is just deal with a day-by-day situation (Hain 2010).  I don’t know anything about it. I don’t want to know. I just take things day by day (Keady 1999). | For the PWD the focus of ‘psychological’ resistance is to fight against developing confusion and uncertainty, and perhaps the fear of becoming a ‘burden’, by holding on to, and presenting, the view that life is continuing normally (Clare 2004). |
|  | Not that I’m shutting out the future completely, I’m aware. I’ve read up about it, heard about it, but I don’t see any sense in letting that colour today (Quinn 2008) |  |
|  | In the end it’s a home for me if I have it . . . Alzheimer’s, my wife won’t cope, I know . . . I don’t blame her. You become a vegetable there . . . no conversation, nothing to do, won’t get out for walks, no exercise. It’s a case of if you don’t use it you lose it. Now you see why I don’t want to know about it.. (Moniz-Cook 2006) | A major concern for the future was the likelihood of increasing isolation, and there was a great deal of worry and uncertainty as to exactly how things would develop (Harman 2006). |
|  | I feel like we are kind of going down this path. It is not even a path; it is like a roller coaster ride. All of sudden you take this huge drop. It even out for a while and you think ok, we are doing all right. Then it takes another dip. You never know when it’s going to happen, when you are going to take the next big slide (Butcher 2001). |  |
|  | If we can’t go dancing . . . have holidays, enjoy together, there is little left for  me . . . maybe her too (Moniz-cook 2006). | Fears about long term consequences of dementia including loss of pleasure (Moniz-cook 2006). |
| **Usefulness or harmfulness of knowledge** | Yes! I read and tear out every little thing you can on the paper. And that helps too a lot of it! . . . So, like to me, if somebody said, ‘Hey something is on about Alzheimer’s.’ I’d drop everything and of course I want to see too. (MacQuarrie 2005)  I’m feeling better recently because the more knowledge you can get about it, that helps just get through the day and to begin to put things back in perspective again (Beard 2008) | Medical knowledge empowering (Beard 2008, Manthorpe 2011) |
|  | It was upsetting to know I could probably look forward to the future where I couldn't control my body functions, and I was hard to get along with.., it was not a cheerful picture, so I just stopped reading all that terrible stuff (Hutchinson 1997) | Receiving a diagnosis of dementia had multiple effects on couples, both positive and negative, and importantly, it did not necessarily increase couples’ understanding and acceptance of what was happening. (Robinson 2005) |
| **Theme 3: Living with dementia** | | |
| **Strategies to minimise or normalise impact of dementia** | I’m trying to control it. Trying to improve on things that I forget about, to improve my memory if I can . . . Appointments . . . I try to write everything down in my diary and look at it (Gillies 2000). | Limitations were noticed but their implications were recontextualized using strategies of minimization and normalization. (MacQuarrie 2005) |
|  | What I’ve done is evolve strategies, with my wife, so that I’m protected against disastrous mistakes (Clare 2002). | The coping strategies that participants described could be viewed as falling along a continuum from self-protective responses serving to maintain a prior or existing sense of self to integrative responses allowing for development and adjustment of the self-concept (Clare 2002). |
|  | We’re a team together . . . become quite smart . . covering up these past years . . . so the children don’t know (Moniz-cook 2006) |  |
|  | It [keys] is on a big bunch and when I go in I throw them on the floor so they are bright green I can’t miss them, they are always there, but if I hang them on a hook, put them in a drawer, on the dresser, in a coat pocket, I panic that I am going to be stuck all day, I can’t go out because I have lost them. But since I throw them there I am all right. (Manthorpe 2011) | People with memory problems tended to develop their own strategies and techniques to manage the impact of the condition on daily life (Manthorpe 2011) |
|  | I find if you have goals in your life, there is always hope. (Duggleby 2009) | Hope seemed to give the participants the physical or mental strength to continue. They continually renewed hope by finding the positives, and seeing possibilities. (Duggleby 2009). |
| **Support from professionals and agencies** | I think because we didn’t know what was available to us we couldn’t use the resources to the best (Lawrence 2008). |  |
|  | It’s nice to meet people and know there in the same boat as you . . . I like coming here, [memory clinic group] I enjoy the company, the nice people and the mix, it’s a break (Wolverson 2010).  [It reminds me that] There’s plenty of other people in the same situation. It’s just a small thing, but it’s very important. And I’m not alone (Beard 2008) | Support groups offered a sense of community but also could be a distressing reminder of what the future might hold (Beard 2008). |
|  | if you work, you get no break… no time to spend with the family alone… and difficulty with transport because you have to get to work on time. Most of these day centres do not open before 9am and that is when I have to be at work (Jutlla 2007). | The data suggests that the barriers present in Asian carers accessing services is largely due to 1) the lack of understanding of dementia which, in turn may lead to 2) failure to seek early professional help, 3) GP misdiagnosis and (or) failure to link to specialist health services. (Jutlla 2007). |
